# The impact of exercise on inflammatory biomarkers: an umbrella review of systematic reviews and meta-analyses

**DOI:** 10.3389/fimmu.2026.1892544

**Published:** 2026-08-25

**Authors:** Wei Zhang, Minghui Du, Zhiyuan Zhang, Yixing Zhan, Longwei Chen, Jie Dong

**Affiliations:** 1 School of Sports and Physical Education, Xi’an Jiaotong University, Xi’an, Shanxi, China; 2 Shanxi Provincial Rehabilitation Hospital, Xi’an, Shaanxi, China

**Keywords:** exercise, inflammatory biomarkers, meta-analyses, systematic review, umbrella review

## Abstract

**Background:**

Chronic low-grade inflammation is a pathophysiological hallmark of various chronic diseases, including cardiovascular diseases, metabolic disorders, and cancers. Regular physical exercise is recognized as an effective strategy to mitigate systemic inflammation; However, existing evidence is primarily derived from systematic reviews focused on specific populations or single biomarkers, resulting in a fragmented evidence base with inconsistent methodological quality. A comprehensive evaluation of the overall quality and certainty of the evidence remains lacking.

**Methods:**

This umbrella review was conducted in accordance with standard guidelines. A systematic literature search was performed in PubMed, Embase, Web of Science, and the Cochrane Library up to January 2026 to identify meta-analyses examining the effects of exercise interventions on inflammatory biomarkers. Two independent reviewers performed literature screening and data extraction. Methodological quality was assessed using the AMSTAR 2 tool, and the certainty of evidence was graded according to the GRADE framework. The review focused on populations with diverse health statuses, providing a comprehensive evaluation of the impact of exercise on multiple inflammatory biomarkers.

**Results:**

A total of 61 meta-analyses were included, encompassing 318 meta-analytic associations (unique exercise-biomarker-population combinations). Of these, 145 (45.6%) showed statistically significant effects of exercise on inflammatory markers. The overall certainty of evidence was generally low: only 10 (3.1%) were rated high quality, 89 (28.0%) moderate quality, and the remaining 219 (68.9%) low or very low quality. Significant heterogeneity was observed in the inflammation-modulating effects of exercise across populations with different health statuses. In most chronic diseases, exercise demonstrated a distinct anti-inflammatory trend. However, in patients with chronic obstructive pulmonary disease and fibromyalgia, exercise may transiently elevate certain inflammatory markers.

**Conclusions:**

This umbrella review suggests that exercise is associated with changes in circulating inflammatory biomarkers across diverse populations, though the certainty of the evidence is generally low to very low. In chronic disease populations, reductions in CRP, IL-6, and TNF-α are generally interpreted as reflecting an attenuation of systemic inflammatory burden, though this interpretation should not be automatically extended to acute post-exercise changes in healthy individuals. Certain exercise modalities showed larger effect sizes in specific populations, but these associations should not be interpreted as evidence of superiority, as the comparisons were indirect, effect sizes may be inflated by the small-study effect, and circulating biomarkers cannot distinguish modality-specific neuromuscular adaptations. Caution is warranted for COPD and fibromyalgia, where exercise may paradoxically trigger acute inflammatory responses. Given the low certainty of evidence and heterogeneity in exercise protocols, the findings should be interpreted as hypothesis-generating rather than definitive, and are intended to inform future research rather than to serve as practice guidelines.

**Systematic review registration:**

https://www.crd.york.ac.uk/PROSPERO/, identifier, CRD420251236842.

## 1 Introduction

Inflammation is a highly conserved physiological process that has evolved to protect the organism against threats such as infection and injury. It is characterized by the orchestrated activation of immune and non-immune cells to eliminate pathogens and promote tissue repair (1, 2). However, when this response loses its normal spatiotemporal regulation and transitions from an acute, self-limiting state to persistent systemic chronic inflammation, it constitutes a common pathological basis for various chronic diseases (3–5). In contemporary society, the global pandemic of physical inactivity has emerged as a key environmental factor exacerbating this pathological process (6). Sedentary behavior is closely associated with obesity, insulin resistance, and visceral fat accumulation (7, 8). These states persistently activate the immune system, releasing pro-inflammatory mediators, thereby establishing and maintaining a harmful state of systemic chronic inflammation (9), which increases the risk of developing diseases such as cardiovascular disease, diabetes, and cancer (6).

According to the American College of Sports Medicine (ACSM), an exercise intervention is defined as any planned, structured, and repetitive physical activity aimed at improving or maintaining physical fitness (10). Substantial evidence indicates that such structured exercise interventions can elicit potent anti-inflammatory effects through multiple mechanisms, including the induction of anti-inflammatory myokines, improvements in body composition and metabolic health, and enhanced immune regulation (11, 12). Therefore, systematically evaluating the impact of exercise interventions on inflammatory biomarkers is of paramount importance for developing precise strategies for chronic disease prevention, and management, as well as health promotion.

To date, numerous systematic reviews and meta-analyses have focused on examining the effects of exercise on specific inflammatory markers—such as C-reactive protein (CRP) and Interleukin-6 (IL-6)—in particular populations, including cardiovascular disease patients and healthy adults. For instance, Yousefabadi et al. (13) reported that exercise significantly improved levels of tumor necrosis factor-alpha (TNF-α), CRP, interleukin-8 (IL-8), and interleukin-10 (IL-10) in patients with metabolic syndrome, concluding that aerobic exercise (AE) alone, or combined with resistance training (RT) represents the optimal modality for this population. In contrast, Guimarães et al. (14) found that exercise may lead to a marginal increase in TNF-α levels in individuals with depression; however, the primary studies on this topic are scarce and likely carry a high risk of bias. Meanwhile, Wang et al. (15) demonstrated that interventions of moderate intensity lasting longer than 12 weeks can significantly reduce inflammatory biomarkers in healthy individuals.

Overall, the evidence in this field is highly fragmented. This fragmentation makes it difficult to delineate the full spectrum of exercise-induced anti-inflammatory effects and hampers comprehensive comparisons across different populations and biomarkers. Crucially, the methodological quality of existing reviews varies considerably, with most failing to systematically assess the certainty of the body of evidence. Without rigorous evidence grading, clinicians and policymakers struggle to evaluate the reliability of these conclusions, which in turn impedes the precise application and promotion of exercise prescriptions in clinical practice.

This umbrella review aims to address these gaps by re-analyzing existing meta-analyses on exercise interventions. We will include studies covering diverse populations (from healthy adults to chronic disease patients) and analyzing exercise’s impact on a wide array of inflammatory biomarkers. By systematically evaluating and grading evidence certainty, this study provides a rigorous, comprehensive synthesis of the current literature. The findings are intended to guide clinical practice, inform public health guideline development, and direct future research.

## 2 Methods

### 2.1 Registration and protocol

This review was conducted as an umbrella review and has been registered in the PROSPERO international prospective register (CRD420251236842; https://www.crd.york.ac.uk/PROSPERO/recorddashboard). This review was prepared in accordance with the Preferred Reporting Items for Overviews of Reviews (PRIOR) statement. The PRIOR checklist is available in **Supplementary Table 1**.

### 2.2 Literature search and screening

Two independent researchers (WZ and ZYZ) conducted a systematic search of four databases -PubMed, Embase, Web of Science, and the Cochrane Library- to identify systematic reviews and meta-analyses examining the relationship between exercise and inflammatory responses. The search covered the period from database inception to January 2026. Detailed search strategies are presented in **Supplementary Table 2**.

Search results were imported into Endnote X9 for management. A dual-investigator screening process was employed: WZ and YXZ independently screened titles and abstracts, followed by full-text review of potentially eligible articles to determine final inclusion in the umbrella review. Any discrepancies were resolved by a third researcher (MHD) through discussion or arbitration to ensure the scientific validity and reliability of the study selection.

### 2.3 Inclusion and exclusion criteria

#### 2.3.1 Inclusion criteria

1. Population: Individuals of all ages and any health status.
2. Intervention: Studies incorporating at least one exercise intervention.
3. Comparison: Exercise intervention groups compared with control groups (usual care, health education, low-intensity physical activity, or no exercise).
4. Outcome: Reporting of at least one inflammatory biomarkers (e.g., CRP, IL-6, TNF-α, IL-1β) and related adipokines (e.g., adiponectin, leptin).
5. Study design: Systematic reviews with meta-analysis.

#### 2.3.2 Exclusion criteria

1. Studies mixing healthy populations with patient populations (If a systematic review reported separate meta-analyses for distinct populations, we extracted the data for each population independently, provided the review’s inclusion criteria were otherwise met. The review was excluded only if it pooled mixed populations without stratification and this pooled result could not be disaggregated).
2. Conference abstracts.
3. Studies combining exercise with other interventions (e.g., diet, lifestyle modification, or physical therapy).
4. Exercise performed under extreme conditions (e.g., hypoxia).
5. Network meta-analyses (Network meta-analyses rely on a statistical framework of indirect comparisons, with effect size estimation and transitivity assumptions that differ fundamentally from those of pairwise meta-analyses. The evidence synthesis framework of this umbrella review was constructed around the frequentist paradigm of pairwise meta-analyses; incorporating evidence from two distinct statistical paradigms into the same evidence synthesis and interpretation framework would compromise the comparability of results and the coherence of interpretation. Therefore, network meta-analyses were not included).

### 2.4 Data extraction

Data extraction from the final eligible studies was performed independently by WZ and MHD. The following data were extracted from each study: first author, publication year, study population, intervention and comparator details, outcome measures, number of included primary studies, sample sizes for experimental and control groups, heterogeneity statistics (I² and Q-test p-value), effect sizes with 95% confidence intervals (CI), and Egger’s test p-value.

Incomplete data were documented, and we attempted to supplement missing values by consulting the original source articles. Data that could not be retrieved were coded to facilitate potential recalculation or sensitivity analysis where feasible.

### 2.5 Quality assessment and evidence grading

The methodological quality of the included systematic reviews with meta-analysis was assessed using the AMSTAR 2 (A Measurement Tool to Assess systematic Reviews) tool, Scientific rigor and applicability (JBI) and Domain-specific risk of bias (ROBIS). AMSTAR 2 has been demonstrated to be a reliable and valid instrument for evaluating the quality of systematic reviews and meta-analyses, encompassing both interventional and observational studies (16, 17). The JBI Checklist for Systematic Reviews and Research Syntheses was used to evaluate the scientific rigor and clinical applicability of the included reviews. Unlike AMSTAR 2, the JBI tool places greater emphasis on the clarity of the review question, appropriateness of inclusion criteria to the research question, adequacy of search strategy sources, independent critical appraisal by two or more reviewers, and the appropriateness of methods for combining studies. It also assesses whether policy/practice recommendations are supported by the reported data. The ROBIS tool was applied to assess the risk of bias across four key domains (1): study eligibility criteria, (2) identification and selection of studies, (3) data collection and study appraisal, and (4) synthesis and findings. ROBIS is specifically designed to evaluate the extent to which flaws in the review process may have biased its conclusions, regardless of the overall reporting quality captured by AMSTAR 2. It provides a domain-level assessment that complements the global quality rating from AMSTAR 2 and the rigor assessment from JBI.

The GRADE (Grading of Recommendations, Assessment, Development and Evaluations) framework was employed to rate the certainty of evidence for each outcome across the body of evidence synthesized in the review (18, 19). In this umbrella review, the GRADE assessment was applied to each unique meta-analytic association (i.e., the pooled result for a specific biomarker in a specific population). Ratings were based on the methodological limitations, inconsistency, indirectness, imprecision, and publication bias as reported by the original systematic review authors or as judged by us from the primary studies cited within the review. Ultimately, the evidence was categorized as being of “High”, “Moderate”, “Low”, or “Very Low” quality.

Additionally, given the inherent practical challenges in blinding participants and personnel within exercise intervention trials, we applied adjusted criteria when assessing the risk of bias related to blinding in randomized controlled trials. Both the quality assessment and evidence grading processes were conducted independently by two researchers (WZ and ZYZ). Any discrepancies were resolved by a third researcher (MHD) to ensure consistency.

### 2.6 Literature categorization

This umbrella review adopted the methodology described by Poole et al. (20) and Huang et al. (21). To minimize evidence overlap, included studies were initially categorized by population type, intervention modality, and comparator. Populations were stratified by health status (e.g., healthy vs. diseased) and specific disease types. Interventions were classified by exercise modality, including AE, RT, high-intensity interval training (HIIT), and other exercise forms. Comparators were primarily grouped into no exercise, usual care, and other exercise-based interventions. To assess primary study overlap across the included meta-analyses, we constructed binary citation matrices for each of the six population subgroups (cardiovascular and respiratory diseases, metabolic diseases, cancer, rheumatic and musculoskeletal diseases, healthy populations, and other chronic inflammation-related populations). We then applied the corrected covered area (CCA) method proposed by Pieper and colleagues (22) to quantify the extent of evidence overlap. According to the classification criteria established in that methodological study, CCA values below 5% indicate slight overlap, values between 5% and 10% indicate moderate overlap, and values above 10% indicate high overlap.

To handle overlapping evidence, we adopted a stratified approach based on the CCA classification. For population subgroups with CCA ≤ 5% (slight overlap), we retained all associations from all included reviews without filtering. For subgroups with CCA > 5%, we applied a hierarchical selection process to control for overlap bias: priority was given to the most recent publication if studies were more than two years apart; for studies published within two years of each other, the one with the larger number of included primary studies was selected; in cases where the number of primary studies was equal, the study with the higher AMSTAR-2 quality rating was prioritized.

### 2.7 Umbrella review analysis method

Due to heterogeneity across meta-analyses regarding population characteristics, intervention/comparator intensity, frequency, and duration, data were extracted exclusively from the pooled results of the included meta-analyses. Given the inherent clinical and methodological heterogeneity across exercise intervention studies, we primarily extracted pooled results from random-effects models. Fixed-effect model results were only used if the original meta-analysis explicitly justified its use based on exceptionally low statistical heterogeneity (I² < 25%) and clinical homogeneity. In practice, this situation did not occur. To address the potential for inappropriate use of fixed-effect models in the included meta-analyses, we prespecified a re-analysis protocol using a random-effects model (23) should such instances be identified. In practice, during data extraction, we carefully examined each included meta-analysis and found no evidence of inappropriate model selection. Accordingly, no re-analysis was required or undertaken.

Evidence was stratified into six categories based on population type: patients with metabolic diseases, patients with cardiovascular or respiratory diseases, cancer patients, patients with musculoskeletal or rheumatic diseases, and healthy individuals and other populations with chronic inflammation-related conditions. Forest plots were generated to visualize the effect of exercise-based interventions on inflammatory responses for each category, displaying effect sizes, population characteristics, sample sizes, heterogeneity (I²), publication bias (Egger’s test p-value), and evidence quality.

To explore whether heterogeneity in control group definitions influenced the observed associations, we conducted a descriptive stratification of the significant findings by control group type. Based on the control group descriptions reported in the original meta-analyses, we classified controls into three categories: (1) non-exercise or no intervention; (2) conventional therapy; and (3) low-intensity exercise. For each population category, we compared the direction and consistency of effect estimates across these control types to assess whether control group heterogeneity materially altered the overall conclusions.

## 3 Result

### 3.1 Literature review

**Figure 1:**
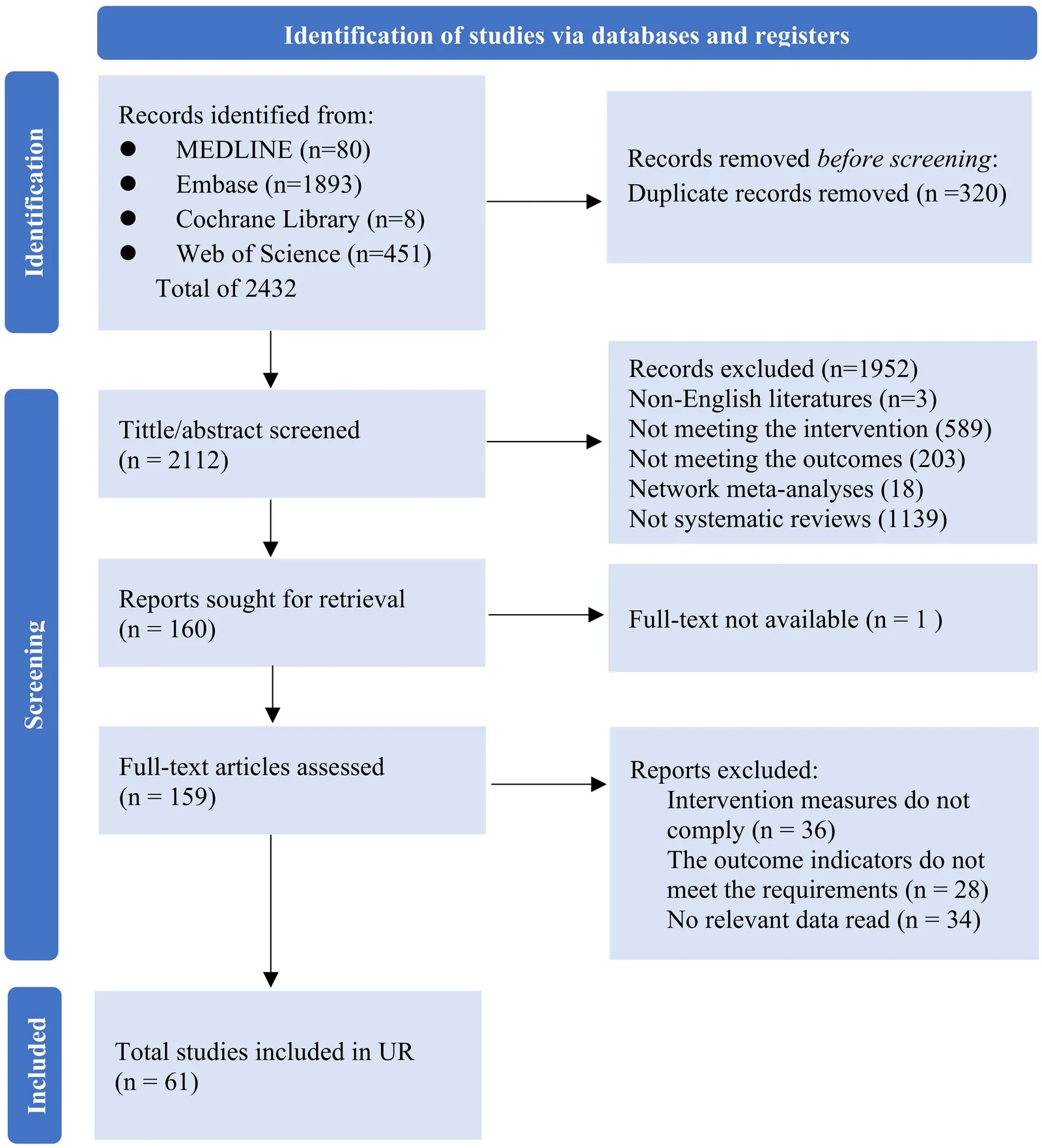
Flowchart of literature screening.

We initially identified 2,437 records from four databases (PubMed, Embase, Web of Science, and Cochrane Library). After removing duplicates and applying screening criteria, 318 meta-analytic associations (unique exercise-biomarker-population combinations) from 61 studies (13–15, 24–81) were included in our umbrella review (**Figure 1**). Subgroup-specific CCA values were 5.58% (cardiovascular and respiratory diseases), 7.3% (cancer), 1.3% (metabolic diseases, healthy populations, and other chronic inflammation-related populations), and 0.85% (rheumatic and musculoskeletal diseases). The full citation matrices are available at Figshare (https://doi.org/10.6084/m9.figshare.33173564).

### 3.2 Characteristics of included meta-analyses and associations

The types of exercise involved in these associations included AE, RT, combined aerobic and resistance training (ART), HIIT, mind–body training (MBT), and mixed exercise. Furthermore, these associations were investigated across populations with different health conditions, including individuals with cardiovascular and respiratory diseases, metabolic disorders, cancer patients, populations with musculoskeletal, rheumatic, and pain-related disorders, as well as healthy and other chronic inflammation-related populations. Among these, 145 associations (45.6%) achieved statistical significance at the p<0.05 level.

### 3.3 Assessment of the methodological quality and evidence certainty

**Figure 2:**
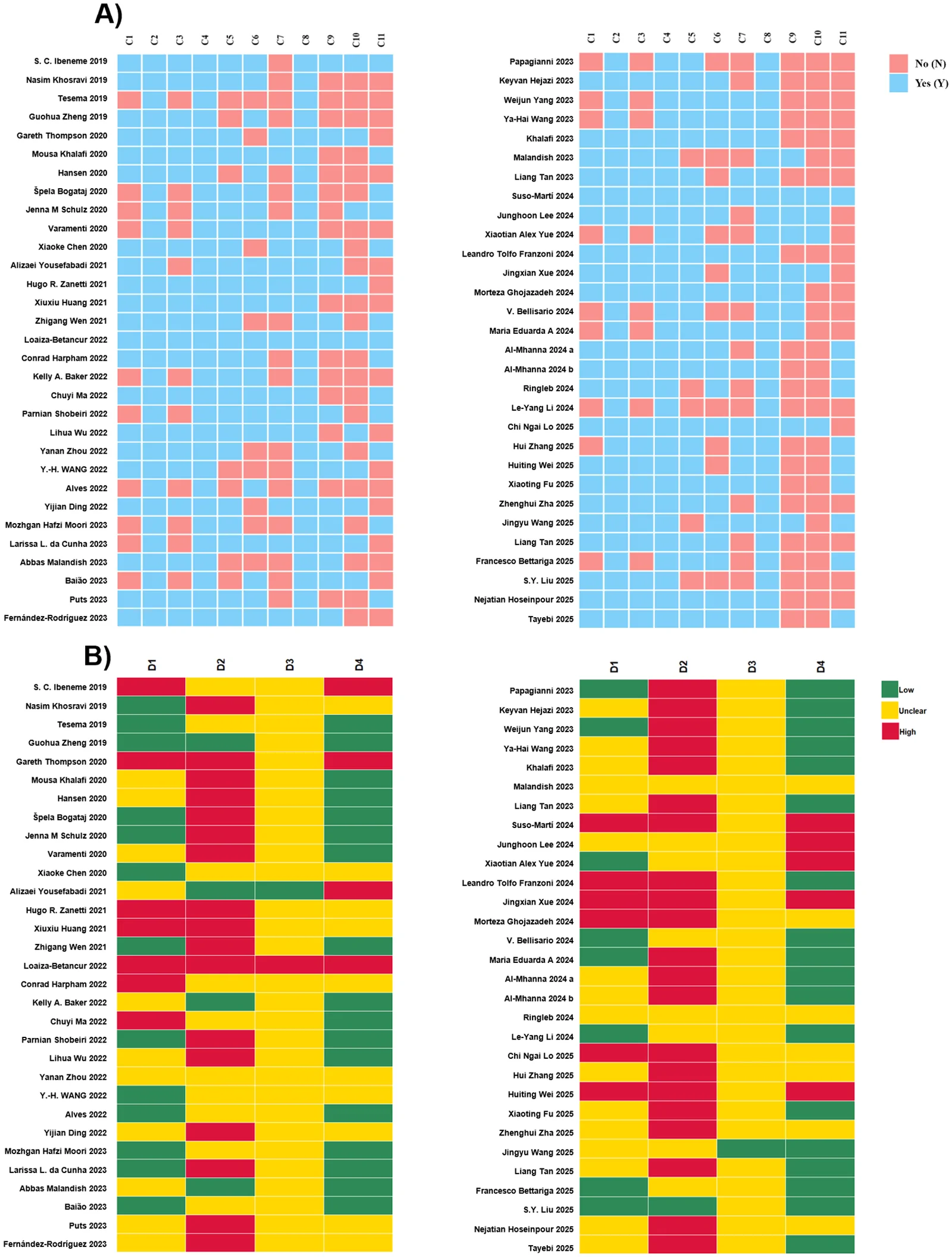
Appraisal results of the JBI and ROBIS tools. **(A)** Results of the JBI appraisal; **(B)** Results of the ROBIS appraisal. Item-Description: C1. Is the review question clearly and explicitly stated? C2. Were the inclusion criteria appropriate for the review question? C3. Was the search strategy appropriate? C4. Were the sources and resources used to search for studies adequate? C5. Were the criteria for appraising studies appropriate? C6. Was critical appraisal conducted by two or more reviewers independently? C7. Were there methods to minimize errors in data extraction? C8. Were the methods used to combine studies appropriate? C9. Was the likelihood of publication bias assessed? C10. Were recommendations for policy and/or practice supported by the reported data? C11. Were the specific directives for new research appropriate? D1. Study eligibility criteria. D2. Identification and selection of studies. D3. Data collection and study appraisal. D4. Synthesis and findings.

AMSTAR results indicate that the overall quality of the 61 included studies is generally low. Specifically, the included meta-analyses lack clear discussion in key areas such as protocol, exclusion lists, and GRADE. Detailed results of the quality assessment are presented in **Table 1**. The JBI assessment results show that 68.85% of the studies clearly articulated the review question; all studies had inclusion criteria appropriate to their research question; 68.85% employed accurate and reliable search strategies; and 68.85% were independently critically appraised by two or more reviewers. Only 24.59% of the studies provided recommendations for policy or practice. Detailed JBI scoring results are shown in **Figure 2A**. The ROBIS results reveal that in the domain of study eligibility criteria, 21 studies were rated as low risk, 27 as unclear, and 13 as high risk. In the domain of study identification and selection, 5 studies were rated as low risk, 18 as unclear, and 38 as high risk. In the domain of data collection and study appraisal, 2 studies were rated as low risk, 58 as unclear, and 1 as high risk. In the “Synthesis and Findings” domain, 9 studies were rated as high risk, 17 as unclear, and 35 as low risk. Details are presented in **Figure 2B**.

**Table 1:** AMSTAR 2 evaluation results of the literature.

Included MA	1	2	3	4	5	6	7	8	9	10	11	12	13	14	15	16
S. C. Ibeneme 2019 (44)	Y	Y	Y	PY	Y	N	N	PY	Y	N	Y	Y	Y	Y	Y	Y
Nasim Khosravi 2019 (47)	Y	N	N	PY	Y	Y	N	PY	Y	N	Y	Y	Y	N	N	Y
Tesema 2019 (67)	N	N	N	PY	Y	N	N	PY	Y	N	Y	N	Y	N	N	Y
Guohua Zheng 2019 (80)	Y	N	N	PY	N	N	N	PY	Y	Y	Y	N	Y	N	N	N
Gareth Thompson 2020 (68)	Y	Y	Y	PY	Y	Y	N	PY	Y	N	Y	Y	Y	Y	Y	Y
Mousa Khalafi 2020 (46)	Y	N	Y	PY	Y	Y	N	PY	Y	N	Y	Y	N	N	N	N
Hansen 2020 (40)	Y	Y	N	PY	Y	N	N	PY	Y	Y	Y	N	N	N	N	Y
Špela Bogataj 2020	N	N	N	PY	Y	Y	N	PY	Y	N	Y	Y	N	N	N	Y
Jenna M Schulz 2020 (61)	N	Y	N	PY	Y	Y	N	PY	Y	Y	Y	N	N	N	N	Y
Varamenti 2020 (69)	N	Y	N	PY	Y	Y	N	PY	Y	N	Y	N	N	Y	N	Y
Xiaoke Chen 2020 (32)	Y	N	N	PY	N	Y	N	PY	Y	Y	Y	N	Y	N	Y	Y
Alizaei Yousefabadi 2021 (13)	Y	N	Y	PY	N	Y	N	PY	N	N	Y	Y	N	Y	Y	Y
Hugo R. Zanetti 2021 (77)	Y	Y	Y	PY	Y	Y	N	PY	Y	N	Y	Y	N	N	Y	N
Xiuxiu Huang 2021 (43)	Y	Y	Y	PY	Y	Y	N	PY	Y	Y	Y	Y	Y	N	N	Y
Zhigang Wen 2021 (73)	Y	N	N	PY	Y	Y	N	PY	Y	N	Y	Y	N	N	Y	N
Loaiza-Betancur 2022 (52)	Y	Y	Y	PY	Y	Y	Y	PY	Y	N	Y	Y	Y	N	Y	N
Conrad Harpham 2022 (41)	Y	Y	Y	PY	Y	N	N	PY	Y	N	Y	Y	Y	N	N	N
Kelly A. Baker 2022 (28)	N	Y	Y	PY	N	N	N	PY	Y	N	Y	Y	N	N	N	N
Chuyi Ma 2022 (53)	Y	Y	Y	PY	N	Y	N	PY	Y	Y	Y	Y	N	N	N	Y
Parnian Shobeiri 2022 (62)	N	N	N	PY	Y	Y	N	PY	Y	Y	Y	Y	N	N	N	Y
Lihua Wu 2022	Y	Y	N	PY	Y	Y	N	PY	Y	Y	Y	N	N	N	N	Y
Yanan Zhou 2022 (81)	Y	Y	N	PY	N	Y	N	PY	Y	N	Y	N	Y	N	Y	Y
Y.-H. WANG 2022 (71)	Y	N	N	PY	Y	N	N	PY	Y	N	Y	Y	N	N	Y	Y
Alves 2022 (26)	N	N	N	PY	Y	N	N	PY	Y	Y	Y	N	Y	N	N	Y
Yijian Ding 2022 (34)	Y	Y	N	PY	Y	Y	N	PY	Y	N	Y	N	Y	N	Y	Y
Mozhgan Hafzi Moori 2023 (39)	N	N	N	PY	Y	N	N	PY	Y	N	Y	N	Y	N	N	N
Larissa L. da Cunha 2023 (33)	N	Y	N	PY	Y	Y	N	PY	Y	N	Y	N	Y	N	NA	Y
Abbas Malandish 2023	Y	Y	N	PY	N	N	N	PY	Y	N	Y	N	N	N	Y	Y
Baião 2023 (27)	N	Y	N	PY	Y	N	N	PY	Y	N	Y	N	Y	N	Y	Y
Puts 2023 (59)	Y	Y	N	PY	Y	Y	N	PY	Y	Y	Y	N	Y	Y	N	Y
Fernández-Rodríguez 2023 (35)	Y	Y	N	PY	Y	Y	N	PY	Y	Y	Y	N	Y	N	Y	Y
Papagianni 2023 (58)	N	N	N	PY	Y	Y	N	PY	Y	N	Y	N	N	N	N	N
Keyvan Hejazi 2023 (42)	Y	Y	N	PY	Y	Y	N	PY	Y	N	Y	N	N	N	N	Y
Weijun Yang 2023 (75)	N	N	N	PY	Y	Y	N	PY	Y	N	Y	N	Y	N	N	Y
Ya-Hai Wang 2023 (15)	Y	Y	N	PY	Y	Y	N	PY	Y	Y	Y	N	N	N	N	Y
Khalafi 2023 (45)	Y	Y	N	PY	Y	Y	N	PY	Y	N	Y	N	N	N	N	Y
Malandish 2023	Y	Y	N	PY	Y	N	N	PY	Y	N	Y	N	Y	N	Y	Y
Liang Tan 2023 (65)	Y	Y	N	PY	Y	Y	N	PY	Y	N	Y	N	N	N	N	Y
Suso-Martí 2024 (63)	Y	Y	Y	PY	Y	Y	N	PY	Y	N	Y	Y	Y	Y	Y	Y
Junghoon Lee 2024 (48)	Y	N	Y	PY	Y	N	N	PY	Y	N	Y	Y	Y	N	Y	N
Xiaotian Alex Yue 2024 (76)	N	N	Y	PY	Y	N	N	PY	Y	N	Y	Y	Y	N	Y	N
Leandro Tolfo Franzoni 2024 (36)	Y	Y	Y	PY	Y	Y	N	PY	Y	Y	Y	Y	N	N	N	Y
Jingxian Xue 2024 (74)	Y	Y	Y	PY	Y	Y	N	PY	Y	N	Y	Y	Y	N	Y	Y
Morteza Ghojazadeh 2024 (38)	Y	Y	Y	PY	Y	Y	N	PY	Y	N	Y	Y	N	Y	N	Y
V. Bellisario 2024 (29)	N	Y	N	PY	Y	N	N	PY	Y	N	Y	N	N	N	N	N
Maria Eduarda A 2024 (14)	N	Y	N	PY	Y	Y	N	PY	Y	N	Y	Y	N	N	N	N
Al-Mhanna 2024 a (24)	Y	Y	N	PY	Y	Y	N	PY	Y	N	Y	N	Y	N	N	N
AL-Mhanna 2024 b (25)	Y	Y	N	PY	Y	Y	N	PY	Y	Y	Y	N	Y	N	N	Y
Ringleb 2024 (60)	Y	Y	N	PY	Y	N	N	PY	Y	Y	Y	N	Y	Y	N	Y
Le-Yang Li 2024 (49)	N	Y	N	PY	Y	N	N	PY	Y	N	Y	N	N	N	N	N
Chi Ngai Lo 2025 (51)	Y	Y	Y	PY	Y	Y	N	PY	Y	N	Y	Y	N	N	Y	Y
Hui Zhang 2025 (79)	N	Y	Y	PY	Y	Y	N	PY	Y	Y	Y	Y	Y	N	N	Y
Huiting Wei 2025 (72)	Y	Y	Y	PY	Y	Y	N	PY	Y	Y	Y	Y	Y	Y	N	Y
Xiaoting Fu 2025 (37)	Y	Y	N	PY	Y	Y	N	PY	Y	Y	Y	N	Y	N	N	Y
Zhenghui Zha 2025 (78)	Y	Y	N	PY	Y	Y	N	PY	Y	N	Y	N	Y	Y	N	Y
Jingyu Wang 2025 (70)	Y	Y	N	PY	Y	N	N	PY	Y	N	Y	N	Y	N	Y	Y
Liang Tan 2025 (64)	Y	Y	N	PY	Y	Y	N	PY	Y	N	Y	N	Y	N	N	Y
Francesco Bettariga 2025 (30)	N	Y	N	PY	N	Y	N	PY	Y	N	Y	N	Y	N	N	Y
S.Y. Liu 2025 (50)	N	N	N	PY	N	N	N	PY	Y	Y	Y	N	N	N	N	Y
Nejatian Hoseinpour 2025 (57)	Y	Y	N	PY	Y	Y	N	PY	Y	N	Y	Y	Y	N	N	Y
Tayebi 2025 (66)	Y	Y	N	PY	Y	Y	N	PY	Y	N	Y	N	N	N	N	Y

ITEM – DESCRIPTION.

1. Did the research questions/inclusion criteria include the components of PICO?

2. Did the review contain an explicit statement that the review methods were established prior to the conduct of the review?

3. Did the review authors explain their selection of the study designs for inclusion in the review?

4. Did the review authors use a comprehensive literature search strategy?

5. Did the review authors perform study selection in duplicate?

6. Did the review authors perform data extraction in duplicate?

7. Did the review authors provide a list of excluded studies and justify the exclusions?

8. Did the review authors describe the included studies in adequate detail?

9. Did the review authors assess the RoB in studies that were included in the review?

10. Did the review authors report on the sources of funding for the studies included in the review?

GRADE evidence revealed that ten of the 318 pieces of evidence were rated as high quality, 89 as moderate quality, 54 as low quality, and 165 as very low quality (**Supplementary Table 3**).

### 3.4 Cardiovascular and respiratory system diseases

**Figure 3:**
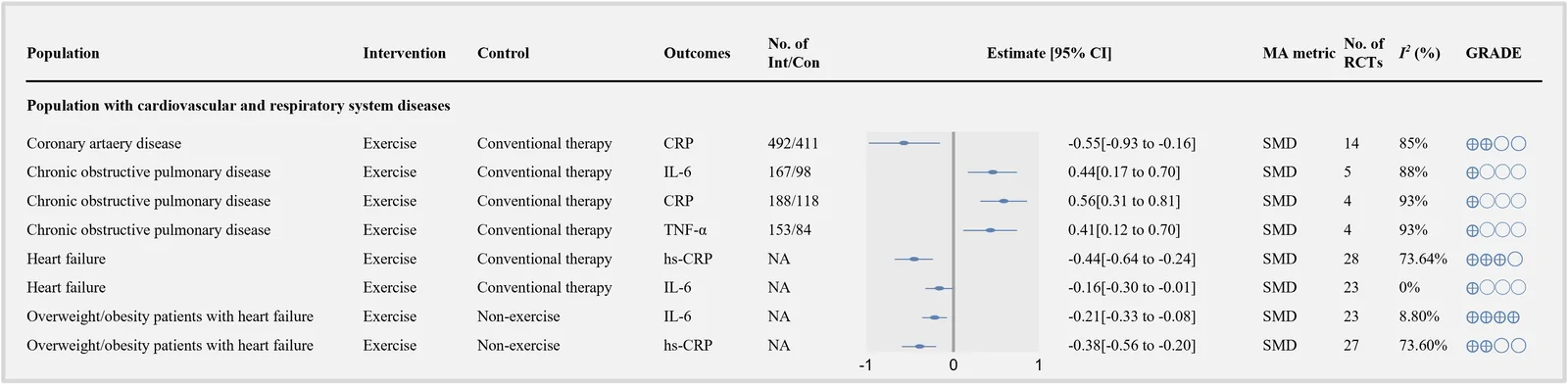
Forest plot of cardiovascular and respiratory system diseases. CI, Confidence interval; Int, Intervention; Con, Control; MA metric, Meta-Analysis metric; GRADE, Grading of Recommendations Assessment, Development, and Evaluation; NA, not available.

We identified eight significant associations between exercise and levels of inflammatory biomarkers in patients with cardiovascular and respiratory diseases, including patients with coronary artery disease (1 association), patients with heart failure (2 associations), patients with heart failure with comorbid obesity/overweight (2 associations), and patients with chronic obstructive pulmonary disease (COPD) (3 associations). Of these, two associations were supported by moderate- to high-certainty evidence (25.0%) (**Figure 3**).

Overall, exercise showed a relatively consistent trend toward reducing inflammatory biomarker levels in patients with heart failure. Moderate-certainty evidence supported that exercise reduced high-sensitivity C-reactive protein (hs-CRP) levels (SMD = −0.44, 95% CI [−0.64 to −0.24]), and high-certainty evidence further confirmed the reducing effect on IL-6 (SMD = −0.21, 95% CI [−0.33 to −0.08]). For patients with coronary artery disease, low-certainty evidence suggested that exercise may reduce CRP levels (SMD = −0.55, 95% CI [−0.93 to −0.16]).

In contrast, patients with COPD exhibited a distinctly opposite pattern. Three very low-certainty evidence pieces indicated that after exercise intervention, their CRP (SMD = 0.56, 95% CI [0.31 to 0.81]), TNF-α (SMD = 0.41, 95% CI [0.12 to 0.70]), and IL-6 (SMD = 0.44, 95% CI [0.17 to 0.70]) levels all showed increasing trends. This paradoxical finding will be discussed in detail in the Discussion section.

To assess whether control group definitions influenced the observed findings, we stratified the eight significant associations in this category by control type. In patients with coronary artery disease and heart failure, the direction of effect was consistently toward reduction of inflammatory markers regardless of whether the control was conventional therapy or non-exercise. In contrast, the three COPD associations all showed elevations in inflammatory markers despite using conventional therapy as the control, suggesting that this paradoxical response is likely attributable to disease-specific pathophysiology rather than control group heterogeneity. However, this stratification was descriptive; the observed differences in effect magnitude between control types were not statistically tested. These observations should therefore be interpreted as descriptive findings, and the potential for effect-size variation to interfere with the accurate estimation of exercise’s true anti-inflammatory effects cannot be ruled out without formal statistical evaluation.

Of note, the overall evidence in this field is relatively scarce, and no study has systematically compared the effects of different exercise modalities on inflammatory markers in patients with cardiovascular or respiratory diseases.

### 3.5 Metabolic diseases

**Figure 4:**
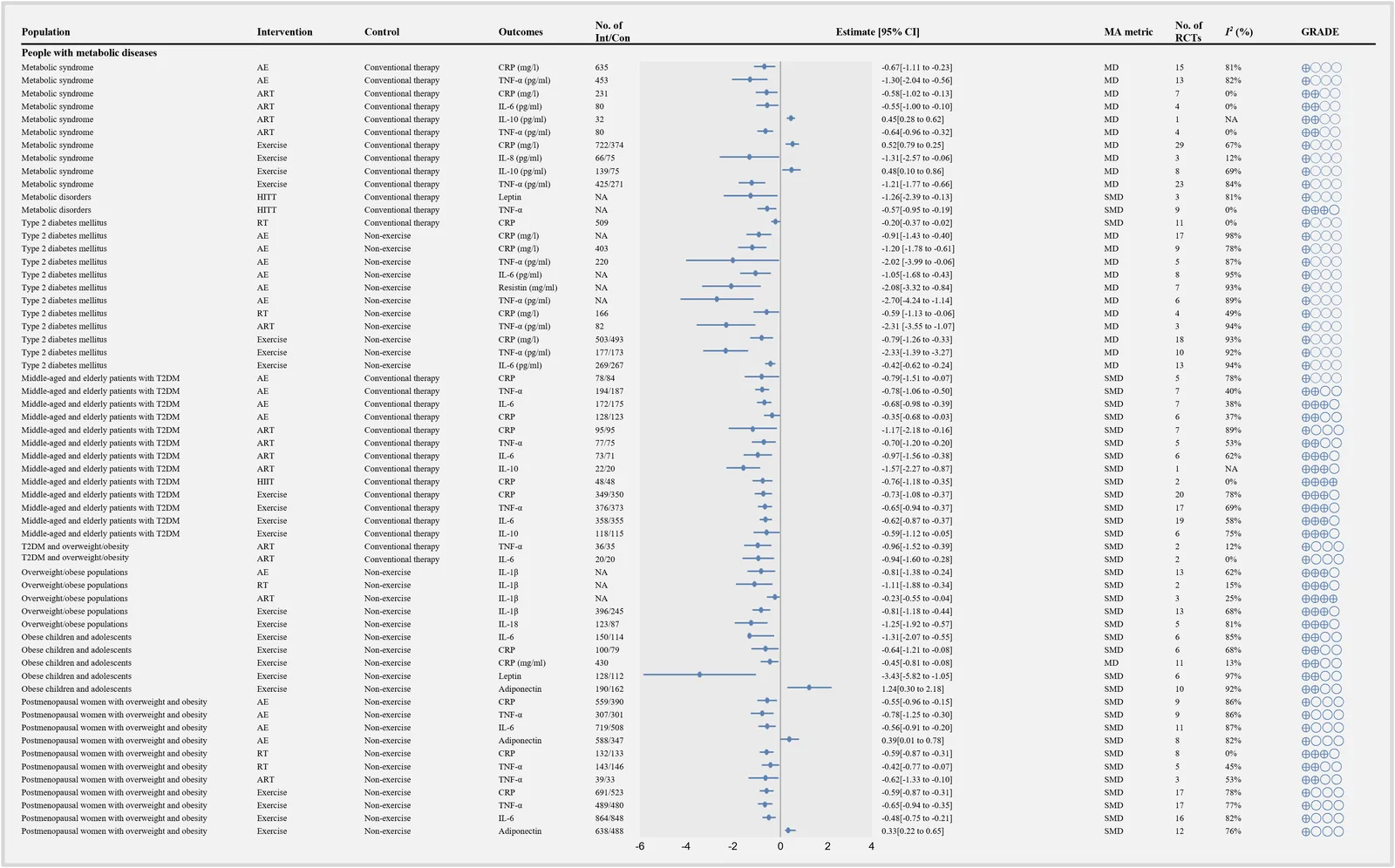
Forest plot of metabolic diseases. CI, Confidence interval; Int, Intervention; Con, Control; MA metric, Meta-Analysis metric; GRADE, Grading of Recommendations Assessment, Development, and Evaluation; NA, Not available; AE, Aerobic exercise; RT, Resistance training; ART, Aerobic combined with resistance exercise; HIIT, High-intensity interval training.

Metabolic disorders represented the most heavily investigated area, with 60 significant associations identified across the following populations: metabolic syndrome (10 associations), metabolic disorder (2), type 2 diabetes (12), middle-aged and older adults with type 2 diabetes (13), type 2 diabetes with comorbid obesity/overweight (2), obesity (5), obese children and adolescents (5), and postmenopausal women with obesity/overweight (11). Of these, 15 associations were supported by moderate- to high-certainty evidence (25.0%) (**Figure 4**). A clear gradient of effect was observed across different exercise modalities in this population.

In terms of exercise modalities, ART was associated with larger effect sizes for reducing CRP, TNF-α, and IL-6 in patients with metabolic syndrome and type 2 diabetes. HIIT showed the largest effect size for improving CRP levels in middle-aged and older adults with type 2 diabetes, supported by high-certainty evidence. In overweight/obese populations, AE, RT, and exercise in general all reduced IL-1β levels, with RT showing a larger effect size (SMD = −1.11).

Regarding inflammatory biomarkers, CRP was the most widely studied marker in metabolic diseases, with exercise associated with its reduction across most subgroups. The associations in middle-aged and older adults with type 2 diabetes (SMD = −0.73) and in individuals with metabolic disorder (SMD = −0.57) were supported by moderate- or high-certainty evidence. Reductions in IL-6 were particularly consistent in patients with type 2 diabetes, with clear effects observed for both ART (SMD = −0.97) and AE (SMD = −0.68). TNF-α reductions were observed in metabolic syndrome, type 2 diabetes, and obesity, although most evidence was of low quality. In children and adolescents with obesity/overweight, exercise was associated with simultaneous reductions in IL-6, CRP, and leptin, as well as increased adiponectin levels, though all relevant evidence was of low certainty. In postmenopausal women with obesity/overweight, multiple evidence pieces supported reductions in CRP, TNF-α, and IL-6, as well as increases in adiponectin, but most were of very low certainty.

In terms of evidence certainty, moderate- to high-certainty evidence was primarily concentrated in middle-aged and older adults with type 2 diabetes (8 associations) and in obese populations (5 associations), whereas most evidence in metabolic syndrome and postmenopausal women with obesity/overweight was rated as low or very low certainty.

To explore whether control group heterogeneity contributed to the observed associations, we stratified the 60 significant findings in metabolic disorders by control type. Across both non-exercise and conventional therapy controls, the direction of effect was remarkably consistent—CRP, IL-6, and TNF-α consistently decreased, while IL-10 and adiponectin consistently increased, irrespective of control group definition. Effect sizes tended to be slightly larger in studies using non-exercise controls than in those using conventional therapy, which is biologically plausible as conventional therapy may include some background health advice. However, because the present analysis was descriptive and did not employ meta-regression or formal statistical tests for subgroup differences, the significance of this effect-size gradient remains uncertain. While the consistency in direction across control types is noteworthy, the magnitude of the observed effects varied considerably, and this variation may significantly interfere with the accurate estimation of the true anti-inflammatory effect of exercise.

Overall, ART was associated with larger effect sizes for inflammatory marker reductions across multiple studies in populations with metabolic disorders. However, given the indirect nature of cross-modality comparisons, the potential inflation of effect sizes by the small-study effect, and the inability of circulating biomarkers to distinguish modality-specific neuromuscular adaptations, this observation should not be interpreted as evidence that ART is superior to other exercise modalities. Given that the majority of the evidence is of low to very low certainty, these findings should be considered exploratory and hypothesis-generating rather than definitive, and further high-quality studies are warranted for confirmation.

### 3.6 Cancer

**Figure 5:**
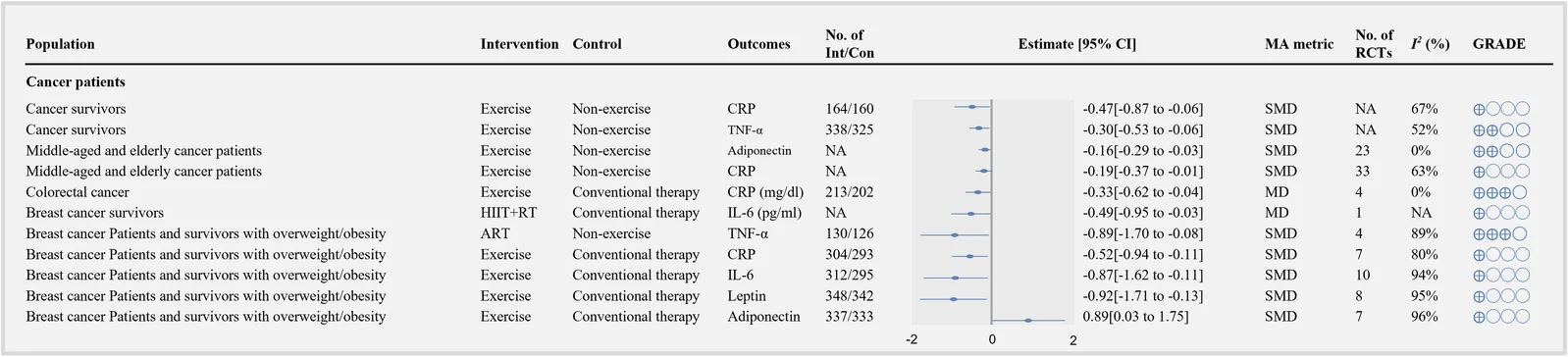
Forest plot of cancer. CI, Confidence interval; Int, Intervention; Con, Control; MA metric, Meta-Analysis metric; GRADE, Grading of Recommendations Assessment, Development, and Evaluation; NA, Not available; ART, Aerobic combined with resistance training; HIIT, High-intensity interval training; RT, Resistance training.

We identified 11 significant associations between exercise and levels of inflammatory biomarkers in cancer populations, covering the following groups: cancer survivors (2 associations), middle-aged and older adults with cancer (2), patients with colorectal cancer (1), patients with breast cancer (1), and patients with breast cancer and comorbid obesity (5). The overall quality of evidence was low, with only 2 associations (18.2%) supported by moderate-certainty evidence (**Figure 5**).

In terms of cancer type, the evidence for colorectal cancer patients was of the highest relative quality, with one piece of moderate-certainty evidence showing that exercise reduced systemic CRP levels (MD = −0.33 mg/dL, 95% CI [−0.62 to −0.04]). Breast cancer patients, particularly those with comorbid obesity/overweight, constituted the most heavily investigated group, with 5 significant associations covering a range of markers including TNF-α, IL-6, CRP, leptin, and adiponectin. Among these, one piece of moderate-certainty evidence supported that ART reduced TNF-α levels (SMD = −0.89, 95% CI [−1.70 to −0.08]), while the remaining four very low-certainty evidence pieces suggested that exercise was associated with reductions in IL-6, CRP, and leptin, as well as increased adiponectin levels. For cancer survivors and middle-aged and older adults with cancer, preliminary evidence suggested reductions in TNF-α and CRP with exercise, although the quality was low to very low.

Regarding evidence certainty, high-quality studies in this field remain scarce, with the majority of significant associations relying on low- or very low-certainty evidence. Large-scale, well-powered randomized controlled trials are urgently needed to clarify the dose-response relationships between exercise type, intensity, and improvements in inflammatory markers. The above findings should be considered exploratory and hypothesis-generating rather than definitive.

To examine the potential impact of control group definitions on the observed associations in cancer patients, we stratified the 11 significant findings by control type. The direction of effect was consistent across non-exercise and conventional therapy controls, with exercise associated with reductions in CRP, IL-6, TNF-α, and leptin, and increases in adiponectin, regardless of the control group used. This stratification was descriptive in nature, and the observed variations in effect size across control types were not subjected to formal statistical testing. Whether these variations meaningfully affect the overall pattern of findings therefore remains uncertain, and the possibility that they may interfere with an accurate estimation of the anti-inflammatory effects of exercise cannot be ruled out without additional analytical approaches.

Overall, the current evidence suggests that exercise may be associated with favorable changes in inflammatory biomarkers in cancer populations, particularly in breast cancer patients with comorbid obesity, but the overall quality of evidence is low. These findings should therefore be interpreted as preliminary and hypothesis-generating, underscoring the urgent need for high-quality, large-scale randomized controlled trials to confirm these associations.

### 3.7 Rheumatic and musculoskeletal diseases

**Figure 6:**
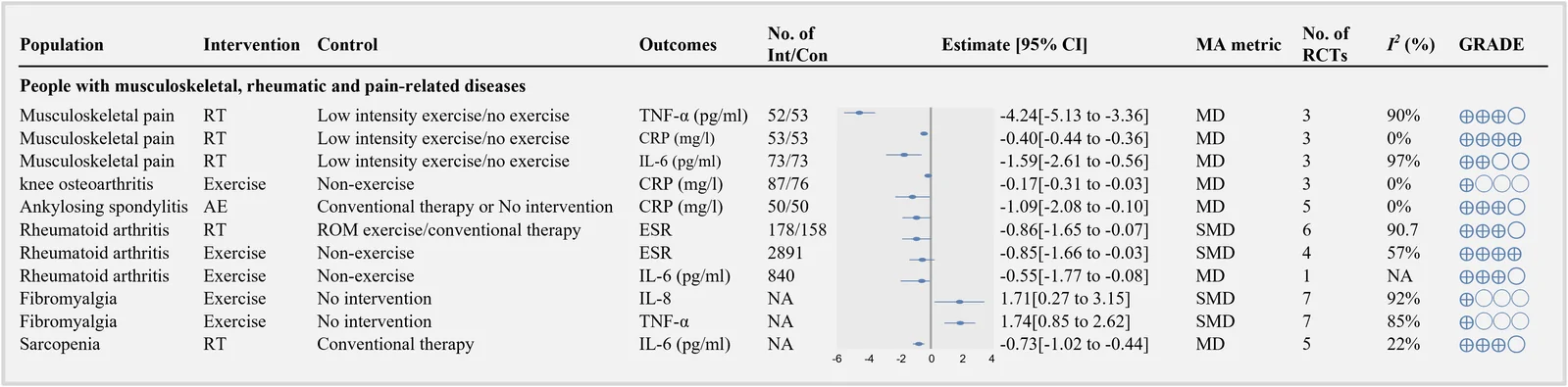
Forest plot of rheumatic and musculoskeletal diseases. CI, Confidence interval; Int, Intervention; Con, Control; MA metric, Meta-Analysis metric; GRADE, Grading of Recommendations Assessment, Development, and Evaluation; NA, Not available; AE, Aerobic exercise; RT, Resistance training; ROM, Range of Motion.

We identified 11 significant associations between exercise and levels of inflammatory biomarkers in populations with rheumatic and musculoskeletal diseases, covering the following subgroups: musculoskeletal pain (3 associations), knee osteoarthritis (1), sarcopenia (1), ankylosing spondylitis (1), rheumatoid arthritis (3), and fibromyalgia (2). Of these, 7 associations (63.6%) were supported by moderate- to high-certainty evidence, making this area one of the highest quality evidence domains in this umbrella review (**Figure 6**).

In terms of exercise modalities, RT was associated with favorable changes in inflammatory markers in this population. In patients with musculoskeletal pain and sarcopenia, moderate- to high-certainty evidence consistently supported that RT significantly reduced CRP, TNF-α, and IL-6 levels. In patients with rheumatoid arthritis (RA), RT showed a larger effect size in reducing erythrocyte sedimentation rate (ESR) compared with other exercise modalities (SMD = −0.86, 95% CI [−1.65 to −0.07]). Additionally, moderate-certainty evidence further supported the reducing effect of exercise on IL-6 in RA patients (MD = −0.55 pg/mL, 95% CI [−1.77 to −0.08]). For patients with ankylosing spondylitis, one piece of moderate-certainty evidence suggested that aerobic exercise reduced CRP levels (MD = −1.09 mg/L, 95% CI [−2.08 to −0.10]).

Regarding inflammatory biomarkers, CRP was the most extensively studied marker in this field, with exercise associated with its reduction across musculoskeletal pain, knee osteoarthritis, ankylosing spondylitis, and RA. Reductions in ESR and IL-6 were primarily observed in RA populations and were supported by high- or moderate-certainty evidence. Notably, RT demonstrated a relatively large effect size in reducing ESR (SMD = −0.86), suggesting its unique advantage in controlling systemic inflammation in RA.

However, patients with fibromyalgia exhibited a distinctly different response pattern. Two very low-certainty evidence pieces showed that exercise paradoxically increased TNF-α (SMD = 1.74, 95% CI [0.85 to 2.62]) and IL-8 (SMD = 1.71, 95% CI [0.27 to 3.15]) levels, a paradoxical finding that will be further explored in the Discussion section.

To evaluate whether control group heterogeneity influenced the observed associations in this category, we stratified the 11 significant findings by control type. The direction of effect was consistent across different control definitions—exercise was associated with reductions in inflammatory markers in patients with rheumatoid arthritis, musculoskeletal pain, ankylosing spondylitis, and sarcopenia, regardless of whether the control was non-exercise, conventional therapy, or low-intensity exercise. The only exception was fibromyalgia, where exercise paradoxically increased TNF-α and IL-8; however, this occurred across studies with no-intervention controls, suggesting this paradoxical response is disease-specific rather than an artifact of control group definition. Nevertheless, the higher certainty of evidence in this subgroup does not itself confer statistical rigor to the control-group stratification, which remained descriptive. Whether the observed effect-size variations across control types reflect meaningful differences or random fluctuation is unclear, and the potential for these variations to distort the accurate estimation of exercise’s anti-inflammatory effects cannot be adequately assessed without meta-regression or other statistical approaches.

Overall, resistance training was associated with favorable changes in inflammatory markers in patients with rheumatic and musculoskeletal diseases, with a higher proportion of evidence rated as moderate or high certainty compared with other disease domains. However, this finding is subject to several methodological limitations: cross-modality comparisons were indirect, observed effect sizes may be inflated by the small-study effect, and circulating biomarkers cannot reflect modality-specific neuromuscular adaptations. Therefore, while these results may provide some reference for clinical practice, they are insufficient to support a definitive conclusion that resistance training is superior to other exercise modalities.

### 3.8 Healthy and other chronic inflammation-related populations

**Figure 7:**
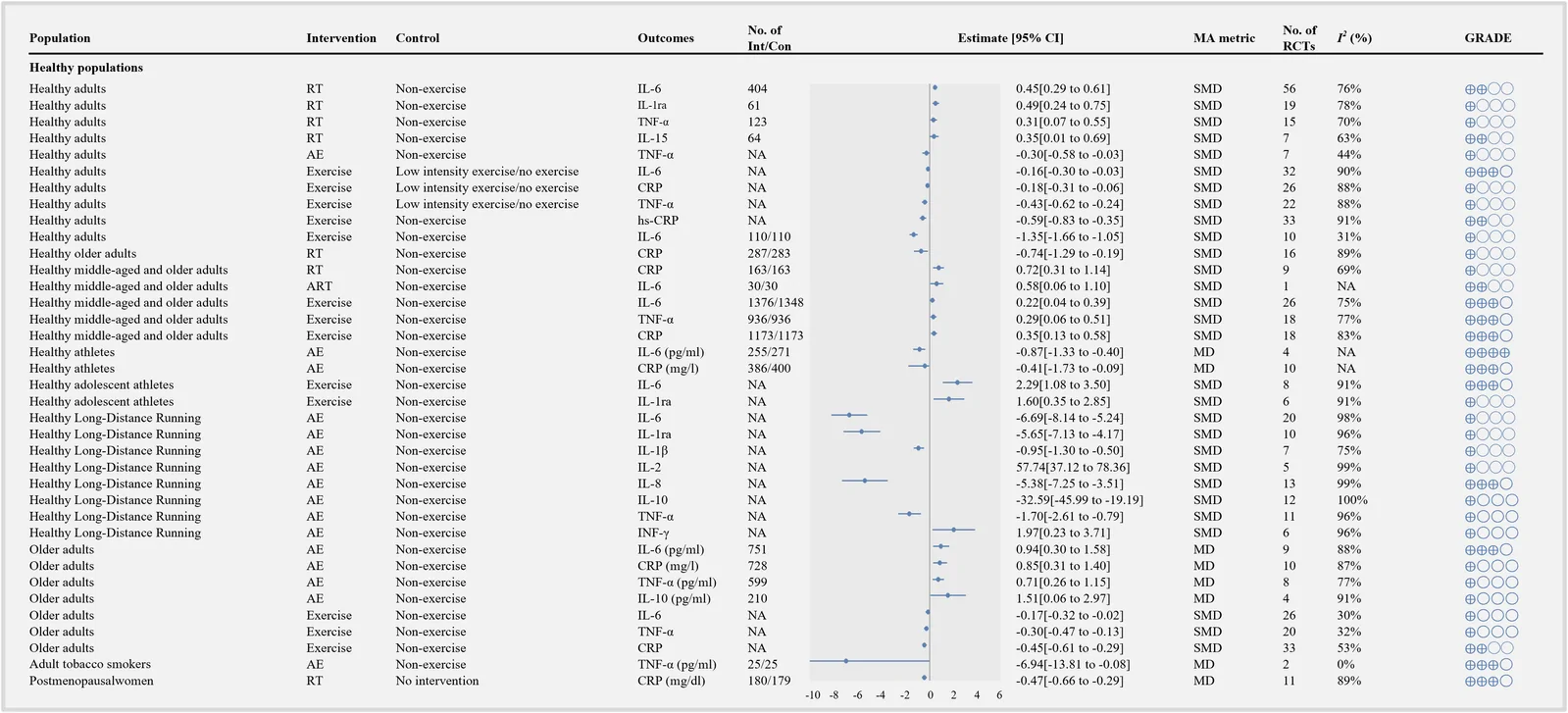
Forest plot of healthy population. CI, Confidence interval; Int, Intervention; Con, Control; MA metric, Meta-Analysis metric; GRADE, Grading of Recommendations Assessment, Development, and Evaluation; NA, Not available; AE, Aerobic exercise; RT, Resistance training.

**Figure 8:**
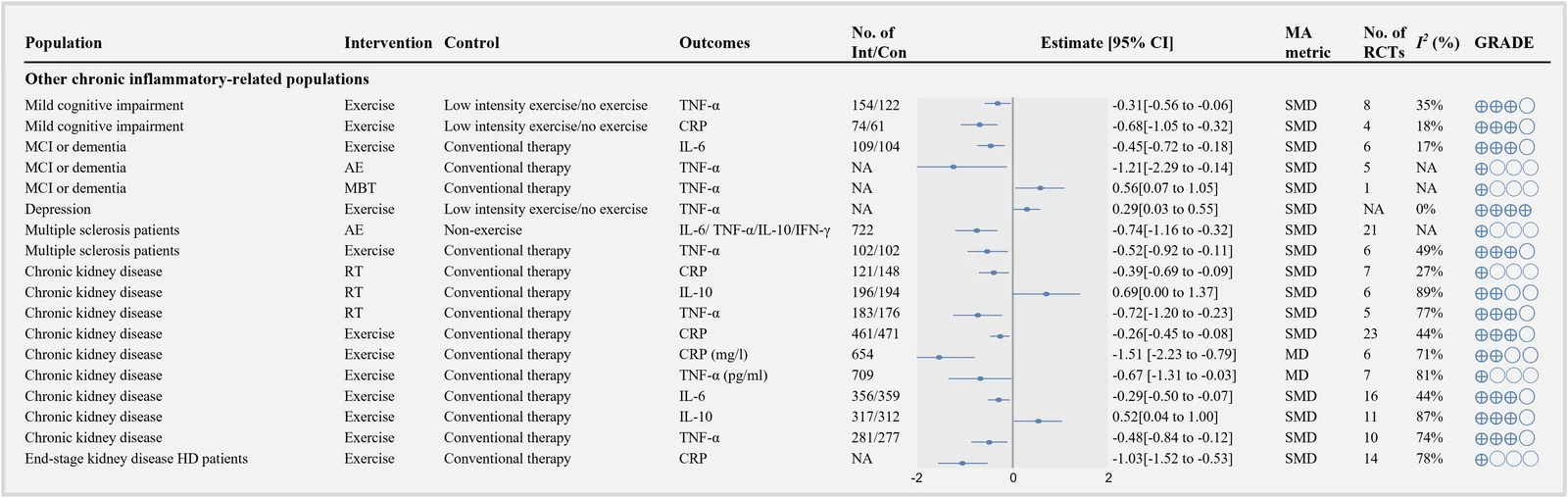
Forest plot of other population with chronic inflammatory-related. CI, Confidence interval; Int, Intervention; Con, Control; MA metric, Meta-Analysis metric; GRADE, Grading of Recommendations Assessment, Development, and Evaluation; NA, Not available; AE, Aerobic exercise; RT, Resistance training.

We identified 55 significant associations between exercise and levels of inflammatory biomarkers in healthy populations and other populations with chronic inflammation-related conditions, covering the following groups: healthy adults (10 associations), healthy middle-aged and older adults (6), healthy athletes (2), healthy adolescent athletes (2), healthy long-distance runners (8), older adults (7), adult smokers (1), postmenopausal women (1), patients with mild cognitive impairment or dementia (5), patients with depression (1), patients with multiple sclerosis (2), and patients with chronic kidney disease (10). Of these, 21 associations were supported by moderate- to high-certainty evidence (38.2%) (**Figures 7**, **8**). The findings in this field exhibited distinct “population-specific” characteristics.

Intensity-gradient effects in healthy populations. In healthy adults, exercise showed a “bidirectional” pattern of inflammatory modulation. On one hand, moderate-certainty evidence indicated that exercise reduced IL-6 levels (SMD = −0.16, 95% CI [−0.30 to −0.03]); on the other hand, multiple very low-certainty evidence pieces showed that acute resistance exercise led to transient elevations in IL-6, IL-15, IL-1ra, and TNF-α. In healthy athletes and long-distance runners, the effect sizes of exercise on inflammatory modulation were generally large, particularly in the substantial reductions of IL-6 (SMD = −6.69), IL-8 (SMD = −5.38), IL-1ra (SMD = −5.65), and TNF-α (SMD = −1.70). However, most of this evidence was of very low certainty, and the elevations in IL-2 (SMD = 57.74) and IFN-γ (SMD = 1.97) in long-distance runners suggested that high-intensity training exerts complex bidirectional effects on immune regulation.

Contradictory patterns in middle-aged and older adults. In healthy middle-aged and older adults, as well as older adults, the evidence was markedly contradictory. Three moderate-certainty studies showed that exercise increased CRP (SMD = 0.35), TNF-α (SMD = 0.29), and IL-6 (SMD = 0.22); conversely, some low- to very low-certainty evidence suggested that exercise reduced these markers. This contradiction may reflect differences between acute exercise stress and long-term training adaptation, as well as inconsistencies across studies in exercise intensity, measurement timing, and baseline participant characteristics.

Response patterns also differed across other chronic inflammation-related populations. In other chronic inflammation-related conditions, the anti-inflammatory effects of exercise exhibited marked “disease-specific” patterns. Moderate- to high-certainty evidence consistently supported that exercise reduced CRP, TNF-α, and IL-6 levels and increased IL-10 levels in patients with chronic kidney disease, with RT showing a larger effect size in reducing TNF-α (SMD = −0.72). Exercise also reduced CRP (SMD = −0.68), TNF-α (SMD = −0.31), and IL-6 (SMD = −0.45) in patients with mild cognitive impairment or dementia, supported by moderate-certainty evidence. However, in patients with depression, moderate-certainty evidence showed that exercise was associated with increased TNF-α levels (SMD = 0.29). For patients with multiple sclerosis, moderate-certainty evidence supported that exercise reduced TNF-α levels (SMD = −0.52).

To assess whether control group heterogeneity affected the observed findings in this heterogeneous category, we stratified the 55 significant associations by control type. Across all subgroups, the direction of effect was generally consistent within each population group regardless of control definition—for example, in healthy athletes and long-distance runners, exercise was associated with reductions in IL-6, IL-8, and TNF-α under non-exercise controls, while in healthy middle-aged and older adults, exercise was associated with increases in inflammatory markers under both non-exercise and low-intensity exercise controls. The consistent directionality within each subgroup, juxtaposed against the stark contrast between subgroups, points to a clear hierarchy: population-level differences (e.g., training status, age, baseline inflammation) exert a dominant influence on exercise-induced inflammatory responses, whereas control group type appears to play a secondary, modulatory role. Nevertheless, because the present stratification was descriptive and did not employ meta-regression, this inference regarding the relative importance of these two sources of heterogeneity remains provisional. The extent to which control group heterogeneity may amplify or obscure the already pronounced population-based variations, and thereby affect the accurate estimation of exercise’s true anti-inflammatory effects, requires formal statistical exploration.

Overall, reductions in circulating inflammatory biomarker levels in healthy populations appeared to be more dependent on exercise intensity and long-term adherence, whereas the direction of effects in middle-aged and older adults remained unclear and contradictory, warranting further high-quality research. For most chronic inflammation-related conditions (e.g., chronic kidney disease, cognitive impairment), long-term regular exercise remains an important strategy for inflammation management; however, the response patterns in conditions such as depression suggest that findings should be interpreted with caution.

## 4 Discussion

### 4.1 Main findings from the umbrella review

This umbrella review systematically synthesizes the existing evidence on the associations between exercise interventions and inflammatory biomarkers across diverse populations. The primary findings is that the impact of exercise on inflammatory biomarkers exhibits significant population heterogeneity.

Overall, in most chronic disease states (e.g., metabolic disorders, cardiovascular diseases, and certain cancers), exercise is associated with reductions in circulating levels of CRP, TNF-α, and IL-6. In these chronic disease settings, where IL-6 is primarily derived from adipose tissue and immune cells and reflects a state of persistent low-grade systemic inflammation, such reductions are generally interpreted as indicative of an attenuation of the chronic inflammatory burden. However, this trend was not universal. In patients with COPD or fibromyalgia, specific exercise modalities led to elevations in certain inflammatory markers. These elevations, however, should not be automatically equated with a worsening of chronic inflammation; they may reflect acute physiological responses to exercise-induced stress (e.g., increased respiratory muscle load in COPD or central sensitization in fibromyalgia) rather than a deleterious shift in the underlying inflammatory state. This highlights the importance of distinguishing between acute exercise responses and chronic training adaptations.

Crucially, the certainty of this evidence is limited. Among the 145 statistically significant associations, only 7 (4.8%) were supported by high-quality evidence, 40 (27.6%) by moderate-quality evidence, and the majority (67.6%) were rated as low or very low quality. This scarcity of high-quality data underscores the need for cautious interpretation and highlights a critical gap in the current literature. To provide a structured framework for this cautious interpretation, it is necessary to adopt a dual-perspective framework when evaluating the findings of this umbrella review. The first perspective is the systemic biomarker response—that is, changes in circulating concentrations of CRP, IL-6, TNF-α, and other inflammatory markers, which constitute the primary evidence base of this review. These circulating biomarkers represent the integrated “spillover” of inflammatory processes occurring across multiple tissues, including adipose tissue, liver, skeletal muscle, vascular endothelium, and synovium. The second perspective is tissue-specific physiological adaptation—that is, the local immunometabolic changes induced by exercise within specific tissues, including altered myokine secretion from skeletal muscle, reduced macrophage infiltration in adipose tissue, improved hepatic insulin sensitivity, and attenuated vascular inflammation. These tissue-level processes constitute the mechanistic pathways that may drive the circulating biomarker changes observed at the systemic level. Within this framework, the findings of this umbrella review primarily provide evidence at the level of systemic biomarker response, systematically describing what happens to circulating inflammatory markers following exercise across different populations. However, circulating biomarkers themselves cannot identify which tissue-specific adaptations are responsible for the observed changes. For example, a reduction in circulating IL-6 following exercise could reflect decreased adipose tissue production, altered skeletal muscle release, reduced hepatic acute-phase response, or any combination thereof—but the systemic measurement itself cannot distinguish among these sources. Therefore, while the observed biomarker patterns are biologically plausible and consistent with known anti-inflammatory mechanisms—such as exercise-induced myokine release, reduced adipose tissue inflammation, and improved insulin sensitivity—they should be viewed as associative evidence that generates hypotheses about underlying mechanisms rather than direct proof of tissue-specific mechanistic improvement.

### 4.2 The dual role of IL-6 and the context-dependent interpretation of inflammatory biomarkers

When interpreting the findings of this umbrella review, a key consideration is the context-dependent biological nature of inflammatory biomarkers, particularly IL-6. In this review, we generally interpret reductions in circulating IL-6 levels as indicative of anti-inflammatory effects. Although this interpretation is reasonable in many chronic disease contexts, it does not fully capture the complexity of IL-6 biology in the exercise setting.

IL-6 is a pleiotropic cytokine that can exert either pro-inflammatory or anti-inflammatory effects depending on its source, timing, and physiological context (82). During acute exercise, contracting skeletal muscle releases substantial amounts of IL-6 into the circulation, often at levels far exceeding those observed in chronic inflammatory diseases (83). This muscle-derived IL-6 is not merely a passive marker of muscle damage; rather, it acts as a metabolically active myokine that stimulates the subsequent release of classical anti-inflammatory cytokines such as IL-1ra and IL-10, while suppressing the production of pro-inflammatory cytokines like TNF-α (84). Thus, in the immediate post-exercise period, transient elevations in IL-6 may reflect the initiation of an anti-inflammatory cascade rather than a harmful pro-inflammatory signal.

Conversely, in the context of chronic diseases—particularly metabolic disorders, cardiovascular disease, and rheumatoid arthritis—persistently elevated basal IL-6 levels are predominantly derived from adipose tissue, macrophages, and other immune cells, and are consistently associated with disease activity, insulin resistance, and adverse clinical outcomes (85). In these settings, reductions in IL-6 levels following regular exercise training typically reflect a genuine attenuation of chronic inflammatory burden.

This “source–timing–context” analytical framework has direct implications for interpreting the findings of this umbrella review. When the included meta-analyses report reductions in IL-6 levels after exercise interventions in chronic disease populations, we interpret these as likely reflecting a meaningful reduction in systemic inflammatory burden. However, when exercise leads to acute elevations in IL-6—as observed in certain healthy athletic populations and specific special groups—these elevations should not be automatically categorized as “pro-inflammatory” or “harmful”. Rather, they may represent normal physiological responses to acute metabolic stress, particularly in the context of high-intensity or prolonged exercise.

The same nuanced interpretation applies to other biomarkers examined in this review. For example, IL-10 is generally regarded as an anti-inflammatory cytokine, but its elevation in certain pathological states does not necessarily indicate a beneficial immune response (86). Adiponectin is typically considered to possess anti-inflammatory and insulin-sensitizing properties, yet its circulating levels are influenced by adipose tissue mass, and its functional activity depends on specific receptor subtypes and target tissues (87). Leptin, in turn, exerts both pro-inflammatory and metabolic regulatory functions, and its interpretation is similarly context-dependent (88).

Furthermore, it is important to recognize that circulating biomarker levels represent systemic “spillover” of various tissue-specific processes. Changes in circulating CRP, IL-6, or TNF-α do not directly reflect local inflammatory events occurring in specific tissues, such as the vascular wall in atherosclerosis, the synovium in rheumatoid arthritis, or the pancreatic islets in type 2 diabetes (89–91). Therefore, while this umbrella review provides a comprehensive summary of systemic biomarker changes in response to exercise, these findings should be viewed as associative evidence rather than direct proof of mechanistic improvement in tissue-specific disease pathways.

In summary, the interpretation of exercise-induced changes in inflammatory biomarkers requires careful consideration of their source, timing, and disease context, rather than simply categorizing biomarkers as uniformly “pro-inflammatory” or “anti-inflammatory”.

### 4.3 The impact of exercise on inflammatory biomarker levels in populations with different health statuses

#### 4.3.1 The modulating effect of exercise on the inflammatory response in populations with metabolic disorders

This umbrella review reveals that exercise exerts a significant regulatory effect on systemic inflammation in populations with metabolic disorders—including metabolic syndrome, type 2 diabetes(T2D), and obesity—supported by relatively robust supporting evidence.

For middle-aged and older adults with type 2 diabetes, high- and moderate-quality evidence from an umbrella review demonstrates that HIIT significantly reduces CRP levels, suggesting a direct reduction in systemic inflammation (92). This finding also implies the potential of HIIT to mitigate insulin resistance in this demographic (93). Beyond HIIT, RT, AE, and ART also lower CRP levels, albeit with lower evidence quality. Notably, ART showed a larger effect size for CRP reduction (SMD = −1.17). Furthermore, moderate-quality evidence from multiple studies shows that both AE and ART significantly reduce IL-6 levels. In the context of type 2 diabetes and other metabolic disorders, persistently elevated basal IL-6 levels—predominantly derived from adipose tissue and immune cells—are closely linked to insulin resistance and diabetic complications (94). The observed reduction in circulating IL-6 is therefore biologically plausible as reflecting decreased adipose tissue IL-6 production—a tissue-specific adaptation. However, this interpretation is inferred from the systemic biomarker pattern rather than directly measured; circulating IL-6 could also reflect changes in hepatic acute-phase response or skeletal muscle release. Thus, while consistent with the hypothesis of reduced adipose tissue inflammation, these findings remain associative at the level of systemic biomarker evidence. Furthermore, it should be noted that this interpretation applies specifically to chronic disease populations with elevated basal IL-6, the same inference should not be automatically extended to acute post-exercise IL-6 elevations in healthy individuals, where IL-6 may serve metabolic signaling functions rather than indicating harm (95). ART demonstrated a larger effect size (SMD = −0.97) specifically in reducing IL-6. Collectively, ART demonstrated larger effect sizes (SMD) in reducing CRP, IL-6, and TNF-α.

In overweight or obese individuals, one high-quality and three moderate-quality associations demonstrated that ART, AE, and exercise in general each significantly reduced IL-1β levels. In obesity, IL-1β secreted by adipose tissue and the liver disrupts insulin signaling pathways, impairing insulin sensitivity in muscle and hepatic tissues (96). This suggests that these exercise modalities may help prevent the progression from obesity to T2D.

For patients with metabolic syndrome, AE reduces their CRP and TNF-α levels (two very low-quality evidence). Evidence from four low-quality studies suggests that ART not only lowers CRP, TNF-α, and IL-6 but also elevates IL-10 levels. This demonstrates the comprehensive regulatory capacity of ART: reducing pro-inflammatory factors while increasing anti-inflammatory cytokines. For metabolic syndrome patients, this represents a distinct advantage.

Overall, aerobic exercise improves cardiorespiratory fitness and reduces visceral fat, which may lower pro-inflammatory adipokine levels, while resistance training stimulates the release of anti-inflammatory myokines (97, 98). ART integrates these two physiological stimuli and was associated with favorable changes in inflammatory markers across multiple studies. However, this observation should not be interpreted as evidence that ART is biologically superior to other exercise modalities. The comparisons across modalities were based on indirect evidence rather than head-to-head direct comparisons. Of particular concern, the original studies in the ART literature generally had small sample sizes, and effect size estimates from small-sample studies tend to be systematically inflated due to greater sampling variability—meaning that the apparent effect-size advantages of ART may, to a considerable extent, reflect statistical artifacts rather than genuine biological effects. Furthermore, circulating biomarkers cannot capture modality-specific neuromuscular adaptations, and thus improvements in inflammatory markers cannot be directly attributed to tissue-specific adaptations unique to ART. In addition, the substantial heterogeneity in exercise protocols across the original studies and the generally low quality of the evidence do not support definitive conclusions regarding optimal exercise prescription.

#### 4.3.2 The regulatory effect of exercise on inflammatory response in individuals with cardiovascular and respiratory diseases

The analysis reveals that exercise exerts a distinct regulatory effect on inflammation in patients with cardiovascular and respiratory diseases; however, the efficacy varies by disease type, with most evidence rated as moderate quality.

For patients with heart failure, moderate-quality evidence demonstrates that exercise is associated with significant reductions in high-sensitivity hs-CRP levels (SMD = -0.44) —a systemic biomarker response consistent with reduced inflammatory tone. However, circulating hs-CRP is a downstream marker of hepatic origin, not a direct measure of myocardial inflammation (99), the observed reduction in hs-CRP does not by itself demonstrate direct attenuation of cardiac tissue inflammation. The observed reduction in hs-CRP therefore provides evidence of reduced systemic inflammatory burden, but does not by itself demonstrate direct attenuation of cardiac tissue inflammation. Furthermore, high-quality evidence confirms that exercise significantly lowers IL-6 levels (SMD = -0.21). In the setting of chronic heart failure, IL-6 is a key mediator of myocardial remodeling and disease progression, with elevated levels predominantly derived from immune cells and cardiac tissue rather than skeletal muscle (100). The observed reduction in circulating IL-6 is consistent with the hypothesis that exercise may attenuate inflammatory drivers of cardiac dysfunction—a tissue-specific adaptation—though the precise mechanisms remain to be fully elucidated, and the systemic measurement cannot distinguish cardiac from other tissue sources.

In contrast, findings for COPD patients are paradoxical. Three very low-quality studies indicate that while exercise reduces CRP levels, it concurrently elevates TNF-α and IL-6. This may be attributed to increased respiratory muscle load and relative hypoxia during exercise, which trigger an acute inflammatory stress response (101). At the molecular level, the increased respiratory muscle load and relative hypoxia during exercise in COPD patients may activate hypoxia-inducible factor-1α (HIF-1α), a key transcriptional regulator that promotes the expression of pro-inflammatory cytokines through the NF-κB pathway (102). This hypoxia-driven inflammatory response, combined with exercise-induced oxidative stress that may temporarily exceed the antioxidant capacity of COPD patients, could explain the transient elevations in TNF-α and IL-6 observed post-exercise. Crucially, these elevations should be interpreted as transient acute responses to exercise-induced physiological stress rather than as evidence of a harmful pro-inflammatory shift in the underlying chronic inflammatory state. This distinction is essential for avoiding the erroneous conclusion that exercise is “detrimental” for COPD patients. Future research must distinguish between acute exercise responses and chronic training adaptations.

For patients with coronary artery disease, low-quality evidence suggests that exercise can lower CRP levels (SMD = -0.55). Inflammatory markers serve as strong independent prognostic predictors, comparable to traditional risk factors such as blood pressure and blood lipids (90). Consequently, a reduction in CRP levels typically signifies a decreased risk of future major adverse cardiovascular events (103). Although the quality of evidence regarding the exercise intervention itself is limited, these findings collectively point to the potential inflammatory-modulating value of exercise for this population.

Overall, exercise offers pronounced inflammatory-modulating benefits for most cardiovascular disease patients, particularly in heart failure management. However, for COPD patients, the risk of exercise-induced acute inflammation necessitates caution. Notably, research in this domain remains scarce, with a lack of systematic discussion on how different exercise modalities affect inflammatory responses. Future studies should prioritize this field to develop detailed, evidence-based guidelines for anti-inflammatory exercise in these populations.

#### 4.3.3 The regulatory role of exercise on inflammatory response in cancer patients

This study reveals that exercise exerts a beneficial effect on the inflammatory status of cancer patients; However, the overall quality of evidence is relatively low, necessitating cautious interpretation.

For patients with colorectal cancer, moderate-quality evidence indicates that exercise significantly reduces CRP levels (MD = -0.33 mg/dl). As a marker of systemic inflammation, a reduction in CRP suggests that exercise helps ameliorate cancer-related chronic inflammatory, potentially exerting a positive influence on prognosis and quality of life (104). However, it does not by itself demonstrate improved tumor-related outcomes, as CRP is a non-specific hepatic-derived acute-phase protein that responds to multiple inflammatory stimuli beyond tumor-associated inflammation.

For breast cancer patients, particularly those with comorbid obesity or overweight, exercise demonstrates multifaceted inflammatory-modulating effects. Moderate-quality evidence shows that ART reduces TNF-α levels. Furthermore, four very low-quality studies suggest that exercise is associated with lower IL-6 and leptin levels and higher adiponectin levels. In the context of obesity-related breast cancer, where chronically elevated IL-6 is primarily derived from adipose tissue and contributes to a pro-tumorigenic inflammatory microenvironment, the observed reductions in IL-6—measured as circulating biomarkers that reflect systemic spillover of adipose tissue processes—may reflect improved adipose tissue function rather than a direct anti-cancer effect. At the tissue-specific level, adiponectin is primarily secreted by adipose tissue and exerts anti-inflammatory and insulin-sensitizing effects (105), its elevation, coupled with reduced leptin, is consistent with the hypothesis that exercise may improve adipose tissue function and partially reverse the obesity-related inflammatory microenvironment (106). Thus, the systemic biomarker pattern is consistent with a plausible tissue-specific adaptation—improved adipose tissue function—though whether this translates into reduced local breast tumor microenvironment inflammation or improved clinical outcomes remains unconfirmed and requires tissue-level investigation. For cancer survivors and middle-aged and older adult cancer patients, exercise can reduce TNF-α and CRP levels, although the evidence is rated as low or very low quality. Nevertheless, these findings support the integration of exercise into comprehensive cancer rehabilitation management to alleviate treatment-related side effects, fatigue, and improve quality of life. Future high-quality RCTs are needed to clarify the dose–response relationship between exercise type, intensity, and inflammatory markers improvements.

Chemotherapy, radiotherapy, or surgical treatments often induce systemic inflammatory responses in cancer patients (107). Although research on the inflammatory-modulating effects of exercise in cancer populations remains limited in both quantity and quality, the overall evidence suggests significant inflammatory-modulating benefits. Particularly for patients with comorbid obesity, exercise appears to yield more pronounced interventional effects.

#### 4.3.4 The modulatory effect of exercise on inflammatory responses in patients with musculoskeletal and rheumatic diseases

This study demonstrates that exercise exerts a positive effect on inflammation control in patients with musculoskeletal and rheumatic diseases. Notably, 50% of the associations were supported by moderate- to high-quality evidence, suggesting that the conclusions are relatively robust.

For patients with RA, two high-quality studies demonstrate that exercise is associated with reductions in CRP and ESR levels, while moderate-quality evidence supports its association with lower interleukin-6 (IL-6) levels. At the systemic biomarker level, these reductions suggest decreased overall inflammatory burden. In RA, a disease driven by persistent autoimmune-mediated inflammation, chronically elevated IL-6 is directly implicated in synovial inflammation, joint destruction, and systemic symptoms (108, 109). At the tissue-specific level, the observed reductions in circulating IL-6 and ESR are biologically consistent with the hypothesis that exercise may suppress synovial inflammatory activity (110). However, circulating IL-6 and ESR represent systemic spillover of inflammatory processes occurring in the synovium and other tissues; they should be interpreted as indirect correlates of disease activity rather than direct measures of local joint inflammation. Future studies directly measuring tissue-specific inflammation are needed to confirm these associations. Additionally, one moderate-quality study showed that RT was associated with a larger effect size for reducing ESR levels (SMD = −0.86), suggesting its potential value in mitigating systemic inflammation in RA patients. Since CRP and ESR are non-specific inflammatory markers, their reduction directly reflects a decrease in the patient’s systemic inflammatory burden (111). RA patients often experience muscle atrophy and loss due to chronic inflammation and limited mobility, a condition referred to as “rheumatoid cachexia (112)”. As skeletal muscle is the largest metabolic and endocrine organ. RT directly increases muscle mass, thereby promoting the release of anti-inflammatory myokines (113). At the tissue-specific level, this mechanism provides biological plausibility for the observed systemic ESR reduction: improved muscle mass and function may contribute to reduced inflammatory burden through enhanced myokine secretion.

For patients with musculoskeletal pain and those with sarcopenia, moderate- to high-quality evidence indicates that resistance exercise can significantly reduce inflammation levels.

Notably, in patients with fibromyalgia, two very low-quality evidence suggest that exercise may paradoxically increase TNF-α and IL-8 levels. This may be attributed to central sensitization, poor exercise tolerance, and potential symptom exacerbation during the initial phase of exercise in fibromyalgia patients (114). From an immunological perspective, the paradoxical elevation of TNF-α and IL-8 in fibromyalgia patients may be linked to central sensitization and hypothalamic-pituitary-adrenal (HPA) axis dysfunction (115). In fibromyalgia, the HPA axis often exhibits blunted cortisol responses to stress, which may impair the normal anti-inflammatory feedback mechanisms (116). Consequently, exercise-induced tissue stress may be amplified rather than dampened, leading to elevated pro-inflammatory cytokine production. It is recommended that gentle, progressive exercise interventions—such as aquatic exercise or MBT—be adopted for this population, with ongoing monitoring of their response.

Overall, resistance training was associated with favorable changes in inflammatory markers in patients with rheumatic and musculoskeletal diseases, with a higher proportion of evidence rated as moderate or high certainty compared with other disease domains. This finding suggests that, for these patients, muscle mass is a key determinant of systemic chronic inflammation and disease activity, and that resistance training may promote the release of anti-inflammatory myokines by increasing muscle mass—providing a physiological basis for its application in this population. However, several methodological limitations should be considered when interpreting this conclusion. The apparent advantages of resistance training over other exercise modalities are based on indirect comparisons rather than head-to-head direct evidence. At the same time, the sample sizes of primary studies in this field are generally small, and effect size estimates may be systematically inflated by the small-study effect; thus, the observed larger effect sizes may, to a considerable extent, reflect statistical artifacts rather than true biological superiority. Furthermore, circulating biomarkers cannot distinguish modality-specific neuromuscular adaptations, and therefore improvements in inflammatory markers cannot be directly attributed to tissue-specific adaptations unique to resistance training. The heterogeneity in exercise protocols and outcome measures across studies also does not support definitive conclusions regarding optimal prescription parameters. Although the evidence in this population is of relatively higher quality, the principle of individualization should still be emphasized—for specific subgroups such as fibromyalgia, exercise tolerance should be more carefully assessed to avoid a “one-size-fits-all” prescription approach.

#### 4.3.5 The regulatory effect of exercise on inflammatory responses in healthy individuals and other chronic inflammation-related populations

The regulatory effect of exercise on inflammation in healthy and special populations exhibits distinct “population-specific” characteristics, with evidence quality predominantly rated as low to moderate, reflecting the complexity of research in this domain.

For healthy adults, moderate-quality evidence indicates that exercise can significantly reduce their IL-6 levels (SMD = -0.16). Another very low-quality evidence suggests a substantial reduction in IL-6 levels among healthy adults, including athletes and military personnel (SMD = -1.35). Although the quality of this evidence is very low, it implies that higher exercise intensity and more rigorous training regimens may enhance the inflammatory-modulating effects of exercise in healthy populations. This observation is supported by two additional moderate-quality and four pieces of very low-quality evidence. These sources propose that AE can reduce levels of IL-8 (SMD = -5.38), IL-6 (SMD = -6.69), IL-1ra (SMD = -5.65), IL-1β (SMD = -0.95), and TNF-α (SMD = -1.70) in healthy athletes. These substantial effect sizes collectively suggest that higher-intensity and more rigorous training regimens are associated with a more favorable systemic inflammatory profile. At the tissue-specific level, chronic training adaptations—including improved muscle oxidative capacity, enhanced myokine secretion, and reduced adipose tissue inflammation—may represent the biological basis underlying these systemic changes, though given the very low certainty of the evidence, these mechanistic inferences remain speculative. Regarding healthy middle-aged and older adults, findings are contradictory. One moderate-quality and three very low-quality evidence indicate that AE is associated with increases in IL-6, CRP, TNF-α, and IL-10 levels. Crucially, these elevations should not be automatically interpreted as a “poor anti-inflammatory effect” or as evidence of harm. In healthy populations with low basal inflammatory levels, transient biomarker elevations following exercise may reflect normal physiological responses to acute metabolic stress—such as muscle-derived IL-6 signaling—rather than a worsening of chronic inflammation. This highlights the importance of distinguishing between acute exercise responses and chronic training adaptations, and of not extrapolating interpretations derived from chronic disease populations to healthy individuals. Conversely, one low-quality and two very low-quality evidence suggest that physical activity reduces CRP, IL-6, and TNF-α levels in older adults, demonstrating a more comprehensive inflammatory-modulating effect. Overall, multi-modal exercise appeared to be associated with favorable changes in a broader range of inflammatory markers in this population, however, due to the low quality of evidence, these findings must be interpreted with caution.

In summary, the systemic inflammatory response to exercise in healthy populations appears to be influenced by exercise intensity and adherence, with higher-intensity training showing larger effect sizes in athletes. However, the effects in healthy middle-aged and older adults remain unclear and contradictory. The apparent advantages of higher-intensity or multi-modal exercise over other modalities should not be interpreted as evidence of superiority, as these comparisons are indirect and based on low- to very-low-certainty evidence. These observations should therefore be considered exploratory.

For most other chronic inflammation-related populations, long-term regular exercise remains a crucial strategy for preventing chronic inflammatory diseases. However, two specific populations warrant particular caution. In patients with mild dementia or cognitive impairment, aerobic exercise appeared to show more consistent associations with reduced inflammatory markers, whereas mind–body training showed a potential pro-inflammatory tendency. This observation is based on indirect comparisons and should not be interpreted as evidence that aerobic exercise is superior to other modalities. In patients with chronic kidney disease, resistance training was associated with a larger effect size for TNF-α reduction, but this apparent advantage arises from indirect comparisons and may be influenced by the small-study effect; it should not be interpreted as evidence that resistance training is superior to other exercise forms. For patients with depression, moderate-quality evidence indicates that exercise is associated with increased TNF-α levels (SMD = 0.29, 95% CI [0.03 to 0.55]). In the context of neuroimmune dysregulation, this elevation may reflect a complex interaction between exercise-induced physiological stress and hypothalamic–pituitary–adrenal (HPA) axis dysfunction, rather than direct evidence of worsening chronic inflammation (117). The biological significance of TNF-α elevation in this setting remains to be further elucidated.

### 4.4 Preliminary observations on exercise prescription for modulating systemic inflammatory biomarkers in populations with different health statuses

The vast majority of patients with metabolic diseases exhibit varying degrees of insulin resistance, characterized by a decreased sensitivity of the body to insulin (118). Insulin resistance promotes a state of chronic low-grade systemic inflammation in patients. This chronic inflammatory state, in turn, exacerbates insulin resistance, creating a vicious cycle that drives disease progression (119). As a potent external intervention, exercise can simultaneously ameliorate insulin resistance and mitigate chronic systemic inflammation, thereby breaking this cycle. Based on the findings of this study, HIIT, AE, and RT were all associated with reductions in chronic inflammation levels in patients with metabolic diseases. Among these, based on indirect comparisons across separate pairwise meta-analyses rather than head-to-head network meta-analyses, ART showed more favorable associations with inflammatory marker reductions in several studies. It is important to emphasize that these comparisons are indirect, and definitive conclusions about the superiority of ART over other modalities cannot be drawn without dedicated network meta-analyses. Given that the majority of this evidence is of low or very low certainty, and that exercise protocols varied considerably across the included meta-analyses, the following suggestions should be interpreted with caution and considered as preliminary observations rather than definitive prescriptions. With this caveat, ART may be considered as a potentially useful strategy for patients with metabolic disorders who are seeking anti-inflammatory benefits through exercise. The defining feature of this exercise regimen is the synergistic integration of AE and RT within a single training session. For elderly patients with metabolic diseases, close monitoring during the intervention is essential to prevent falls. For obese/overweight patients, careful management of knee joint loading is required to avoid excessive mechanical stress caused by body weight. Furthermore, analysis of the original RCTs reveals that the predominant ART training schedule was 3–4 sessions per week, with each session lasting 60–90 minutes. The aerobic component was performed at an intensity of 65–70% of maximum heart rate (HRmax), while the resistance exercise was conducted at an intensity of 70% to 85% of one-repetition maximum (1RM). It is important to note, however, that these parameters were derived from descriptive summaries of the original RCTs and were not uniformly applied across all included meta-analyses. Considerable heterogeneity exists in exercise prescription protocols, and these values should not be interpreted as universally recommended doses. Future studies specifically designed to compare different exercise dosages are needed to establish optimal prescription parameters.

For patients with rheumatic and musculoskeletal diseases, resistance exercise may be of particular interest based on the available evidence. However, it should be noted that the evidence supporting this observation, while of relatively higher quality compared to other populations, still originates from a limited number of studies with heterogeneous outcome measures. Most individuals with rheumatic and musculoskeletal disorders experience chronic inflammatory processes (120). The persistent release of inflammatory cytokines leads to excessive osteoclast activation and concurrent impairment of osteoblast function, resulting in compromised bone integrity (121). In these patient populations, physical activity often induces significant pain, leading patients to reduce movement to avoid the onset or exacerbation of discomfort; Consequently, muscle mass decreases markedly. Resistance exercise promotes the production of myogenic anti-inflammatory factors through repeated muscle contractions, thereby modulating disease activity. Simultaneously, it increases muscle mass and enhances periarticular muscle strength, which mitigates joint stress and structural damage, further alleviating pain. Such patients require additional encouragement and support during exercise to improve their adherence. Moreover, given the poor exercise tolerance in individuals with fibromyalgia, a gentler, progressive exercise regime should be adopted, with close attention paid to the patient’s subjective experience and timely adjustment of intensity. We note that numerous primary studies employed resistance exercise interventions conducted 3 to 4 times per week, each session lasting 30 to 60 minutes at an intensity of 70% to 90% of 1RM. It should be noted, however, that these parameters were derived from descriptive summaries of the original RCTs and were not uniformly applied across all included meta-analyses. Furthermore, optimal prescription parameters may vary considerably depending on disease type, baseline disease activity, and functional limitations.

**Figure 9:**
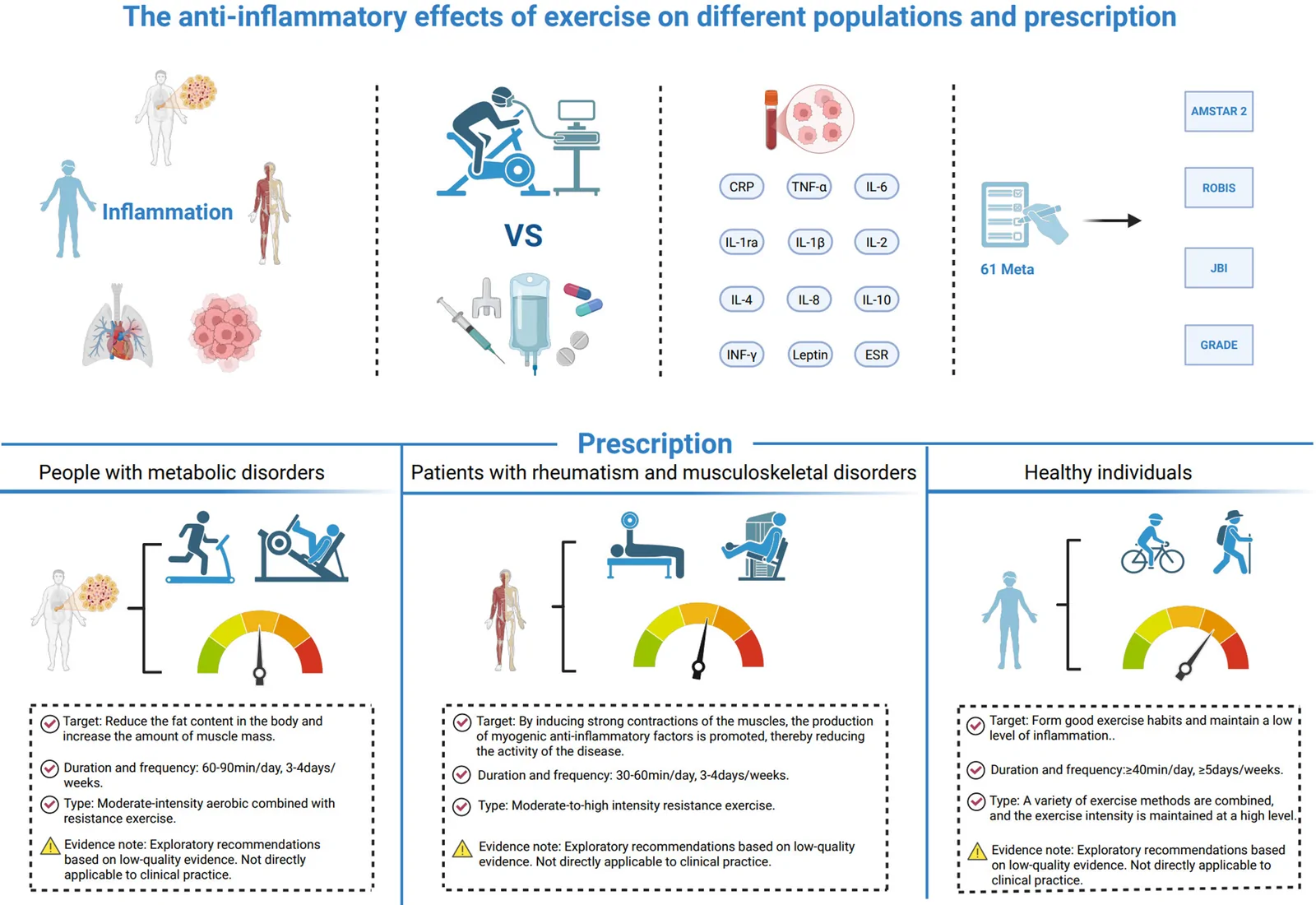
Visualization of the main findings from the umbrella review and exercise prescription recommendations for different populations.

For healthy populations, a wider range of exercise modalities is available, and their inflammatory-modulating effects are inextricably linked to exercise intensity and adherence. In contemporary society, sedentary behavior combined with inadequate sleep often places healthy individuals in a pro-inflammatory state (122). Findings from this umbrella review indicate that AE alone does not yield superior inflammatory-modulating effects compared to mixed-form exercise regimens. Moreover, athletes and military personnel engaging in long-term high-intensity, high-frequency training exhibit lower levels of chronic inflammation. Therefore, based on the available evidence, it appears reasonable to suggest that healthy individuals may benefit from actively participating in diverse forms of physical activity, maintaining at least moderate intensity, and cultivating a habit of regular exercise. However, given the generally low quality of evidence in this population, these suggestions should be considered preliminary and subject to revision as higher-quality evidence emerges. The main findings of UR and the recommended exercise prescription are shown in **Figure 9**.

### 4.5 Limitations and future research directions

This study has several limitations. First, the quality of the included meta-analyses varies, and the original studies predominantly featured small to moderate sample sizes. This results in an overall low certainty of evidence, particularly given the high proportion of evidence of very low quality. Second, at the review level, conducting detailed subgroup analyses regarding exercise dosage (frequency, intensity, time, and type) posed challenges, which restricts the derivation of optimal exercise prescriptions. This limitation is particularly important because it directly restricts the derivation of optimal exercise prescriptions. Consequently, the exercise suggestions provided in this review should be regarded as preliminary and exploratory rather than prescriptive; they are intended to guide future hypothesis-driven research rather than to serve as definitive clinical practice guidelines.

Furthermore, the conclusions of this review are based exclusively on circulating inflammatory biomarkers—a practical and widely used approach that enables synthesis of evidence across diverse populations and exercise modalities. However, as emphasized throughout the preceding sections, these conclusions must be interpreted within a broader biological context to fully appreciate both the value and the limitations of this approach. Circulating biomarkers provide a valuable, non-invasive window into systemic inflammatory status. They are clinically accessible, standardized, and consistently associated with disease risk and prognosis in epidemiological studies. In exercise research, they allow comparisons across populations, exercise modalities, and intervention durations.

Nevertheless, circulating biomarkers reflect systemic “spillover” of inflammatory processes occurring in various tissues—including adipose tissue, liver, skeletal muscle, vascular endothelium, and synovium—without identifying the specific tissue source or the nature of the underlying pathology. For example, a reduction in circulating CRP does not distinguish between decreased hepatic inflammation, improved adipose tissue function, or attenuated vascular inflammation. Similarly, a reduction in circulating IL-6 does not reveal whether it reflects decreased adipose tissue production (generally beneficial in chronic disease) or altered skeletal muscle release (which may carry different metabolic implications). Importantly, the biomarker patterns observed in this review are biologically plausible in light of established mechanistic pathways: exercise promotes the release of anti-inflammatory myokines (e.g., muscle-derived IL-6, which induces IL-1ra and IL-10), reduces adipose tissue mass and macrophage infiltration, and improves insulin sensitivity—all of which may contribute to reduced circulating inflammatory marker levels. However, these mechanistic pathways are inferred from the observed biomarker patterns rather than directly measured in this review. In summary, this umbrella review provides robust evidence that exercise is associated with changes in circulating inflammatory biomarkers across diverse populations. These findings are consistent with—and generate hypotheses about—the anti-inflammatory mechanisms of exercise, but they do not by themselves constitute direct proof of tissue-specific mechanistic improvement or clinical benefit.

In addition, network meta-analyses were not included in this umbrella review. Network meta-analyses rely on a statistical framework of indirect comparisons, with effect size estimation and transitivity assumptions that differ fundamentally from those of pairwise meta-analyses. Incorporating evidence from two distinct statistical paradigms into the same review system would compromise the comparability of results and the coherence of interpretation. Therefore, we chose to restrict our analysis to pairwise meta-analyses. While this decision ensured methodological homogeneity, it also limited our ability to directly compare the relative effects of different exercise modalities—an area in which network meta-analyses offer particular advantages in ranking comparative effectiveness. Accordingly, the exercise recommendations involving comparisons across different modalities in this review should be interpreted as preliminary observations based on indirect comparisons rather than definitive conclusions.

Future research should prioritize conducting more large-scale, long-term follow-up, and methodologically rigorous randomized controlled trials to provide higher-quality evidence. Furthermore, future studies should also emphasize investigating the dose-response relationship of exercise to establish the minimum effective volume and optimal intensity required to achieve the best anti-inflammatory outcomes for different populations. Furthermore, to overcome the inherent limitations of circulating biomarker measurements, future research should incorporate tissue-specific assessments—such as adipose tissue biopsies, skeletal muscle gene expression analyses, and imaging techniques to evaluate vascular and synovial inflammation. Integrating these tissue-level measurements with circulating biomarker data would provide a more complete understanding of the mechanistic pathways through which exercise exerts its inflammatory-modulating effects, and would help distinguish between systemic spillover and genuine tissue-specific improvements.

## 5 Conclusions

This umbrella review systematically consolidates synthesized existing systematic reviews and meta-analyses to comprehensively assess the impact of exercise interventions on inflammatory biomarkers across diverse populations. In chronic disease populations, exercise is associated with reductions in circulating levels of core inflammatory markers such as CRP, IL-6, and TNF-α, which are generally interpreted as reflecting an attenuation of systemic inflammatory burden in these settings. However, this interpretation should not be automatically extended to acute post-exercise changes in healthy individuals, where transient elevations in IL-6 may represent physiological metabolic signaling rather than harm. Although certain exercise modalities—particularly ART in metabolic diseases and RT in musculoskeletal and rheumatic diseases—showed larger effect sizes in specific populations, these comparisons were indirect and should not be interpreted as evidence of superiority. The apparent effect-size advantages may be inflated by the small-study effect, and the systemic nature of circulating biomarkers precludes attribution of changes to modality-specific neuromuscular adaptations. However, caution is warranted for specific populations, including those with chronic obstructive pulmonary disease and fibromyalgia, as exercise may paradoxically trigger acute inflammatory responses, this underscores the necessity for strictly individualized prescriptions and close clinical monitoring. Given the generally low-to-very-low certainty of the evidence and the substantial heterogeneity in exercise protocols across the included meta-analyses, the exercise suggestions provided in this review should be interpreted as hypothesis-generating rather than definitive clinical prescriptions. They are intended to inform future research and clinical investigation, not to serve as practice guidelines. Future research should prioritize large-scale, long-term, and methodologically rigorous randomized controlled trials to definitively clarify the dose-response relationships between exercise parameters (type, intensity, frequency, and duration) and anti-inflammatory outcomes.
