# Supplementary material for: The impact of exercise on inflammatory biomarkers: an umbrella review of systematic reviews and meta-analyses
Source: Front Immunol. 2026 Aug 25;17:1892544. doi: 10.3389/fimmu.2026.1892544 (PMC13552411; doi:10.3389/fimmu.2026.1892544)
Supplement: Supplementary file 1 [file Table1.docx]

**Supplementary Materials**

**Content**

**Table S1** PRIOR checklist

**Table S2** Search strategy

**Table S3** GRADE classification of quality of evidence

**Table S2** PRIOR checklist (preferred reporting items for overviews of reviews)

| Section topic | Item No | Item | Location where item is reported |
| --- | --- | --- | --- |
| **Title** | | |  |
| Title | 1 | Identify the report as an overview of reviews. | 1 |
| **Abstract** | | |  |
| Abstract | 2 | Provide a comprehensive and accurate summary of the purpose, methods, and results of the overview of reviews. | 2-3 |
| **Introduction** | | |  |
| Rationale | 3 | Describe the rationale for conducting the overview of reviews in the context of existing knowledge. | 3-4 |
| Objectives | 4 | Provide an explicit statement of the objective(s) or question(s) addressed by the overview of reviews. | 4 |
| **Methods** | | |  |
| Eligibility criteria | 5a | Specify the inclusion and exclusion criteria for the overview of reviews. If supplemental primary studies were included, this should be stated, with a rationale. | 5 |
|  | 5b | Specify the definition of “systematic review” as used in the inclusion criteria for the overview of reviews. | 5 |
| Information sources | 6 | Specify all databases, registers, websites, organisations, reference lists, and other sources searched or consulted to identify systematic reviews and supplemental primary studies (if included). Specify the date when each source was last searched or consulted. | 4 |
| Search strategy | 7 | Present the full search strategies for all databases, registers and websites, such that they could be reproduced. Describe any search filters and limits applied. | 4 |
| Selection process | 8a | Describe the methods used to decide whether a systematic review or supplemental primary study (if included) met the inclusion criteria of the overview of reviews. | 4 |
|  | 8b | Describe how overlap in the populations, interventions, comparators, and/or outcomes of systematic reviews was identified and managed during study selection. | 6 |
| Data collection process | 9a | Describe the methods used to collect data from reports. | 5 |
|  | 9b | If applicable, describe the methods used to identify and manage primary study overlap at the level of the comparison and outcome during data collection. For each outcome, specify the method used to illustrate and/or quantify the degree of primary study overlap across systematic reviews. | 6-7 |
|  | 9c | If applicable, specify the methods used to manage discrepant data across systematic reviews during data collection. | 4 |
| Data items | 10 | List and define all variables and outcomes for which data were sought. Describe any assumptions made and/or measures taken to identify and clarify missing or unclear information. | 5 |
| Risk of bias assessment | 11a | Describe the methods used to assess risk of bias or methodological quality of the included systematic reviews. | 6 |
|  | 11b | Describe the methods used to collect data on (from the systematic reviews) and/or assess the risk of bias of the primary studies included in the systematic reviews. Provide a justification for instances where flawed, incomplete, or missing assessments are identified but not reassessed. | 6 |
|  | 11c | Describe the methods used to assess the risk of bias of supplemental primary studies (if included). | NA |
| Synthesis methods | 12a | Describe the methods used to summarise or synthesise results and provide a rationale for the choice(s). | 7 |
|  | 12b | Describe any methods used to explore possible causes of heterogeneity among results. | 7-8 |
|  | 12c | Describe any sensitivity analyses conducted to assess the robustness of the synthesised results. | NA |
| Reporting bias assessment | 13 | Describe the methods used to collect data on (from the systematic reviews) and/or assess the risk of bias due to missing results in a summary or synthesis (arising from reporting biases at the levels of the systematic reviews, primary studies, and supplemental primary studies, if included). | 6 |
| Certainty assessment | 14 | Describe the methods used to collect data on (from the systematic reviews) and/or assess certainty (or confidence) in the body of evidence for an outcome. | 6 |
| **Results** | | |  |
| Systematic review and supplemental primary study selection | 15a | Describe the results of the search and selection process, including the number of records screened, assessed for eligibility, and included in the overview of reviews, ideally with a flow diagram. | 8 |
|  | 15b | Provide a list of studies that might appear to meet the inclusion criteria, but were excluded, with the main reason for exclusion. | NA |
| Characteristics of systematic reviews and supplemental primary studies | 16 | Cite each included systematic review and supplemental primary study (if included) and present its characteristics. | 8 |
| Primary study overlap | 17 | Describe the extent of primary study overlap across the included systematic reviews. | 8 |
| Risk of bias in systematic reviews, primary studies, and supplemental primary studies | 18a | Present assessments of risk of bias or methodological quality for each included systematic review. | 8-9 |
|  | 18b | Present assessments (collected from systematic reviews or assessed anew) of the risk of bias of the primary studies included in the systematic reviews. | 6 |
|  | 18c | Present assessments of the risk of bias of supplemental primary studies (if included). | NA |
| Summary or synthesis of results | 19a | For all outcomes, summarise the evidence from the systematic reviews and supplemental primary studies (if included). If meta-analyses were done, present for each the summary estimate and its precision and measures of statistical heterogeneity. If comparing groups, describe the direction of the effect. | 9-15 |
|  | 19b | If meta-analyses were done, present results of all investigations of possible causes of heterogeneity. | NA |
|  | 19c | If meta-analyses were done, present results of all sensitivity analyses conducted to assess the robustness of synthesised results. | NA |
| Reporting biases | 20 | Present assessments (collected from systematic reviews and/or assessed anew) of the risk of bias due to missing primary studies, analyses, or results in a summary or synthesis (arising from reporting biases at the levels of the systematic reviews, primary studies, and supplemental primary studies, if included) for each summary or synthesis assessed. | 8-9 |
| Certainty of evidence | 21 | Present assessments (collected or assessed anew) of certainty (or confidence) in the body of evidence for each outcome. | 9 |
| **Discussion** | | |  |
| Discussion | 22a | Summarise the main findings, including any discrepancies in findings across the included systematic reviews and supplemental primary studies (if included). | 15 |
|  | 22b | Provide a general interpretation of the results in the context of other evidence. | 15-21 |
|  | 22c | Discuss any limitations of the evidence from systematic reviews, their primary studies, and supplemental primary studies (if included) included in the overview of reviews. Discuss any limitations of the overview of reviews methods used. | 21-22 |
|  | 22d | Discuss implications for practice, policy, and future research (both systematic reviews and primary research). Consider the relevance of the findings to the end users of the overview of reviews, eg, healthcare providers, policymakers, patients, among others. | 20-21 |
| **Other information** | | |  |
| Registration and protocol | 23a | Provide registration information for the overview of reviews, including register name and registration number, or state that the overview of reviews was not registered. | 4 |
|  | 23b | Indicate where the overview of reviews protocol can be accessed, or state that a protocol was not prepared. | 4 |
|  | 23c | Describe and explain any amendments to information provided at registration or in the protocol. Indicate the stage of the overview of reviews at which amendments were made. | NA |
| Support | 24 | Describe sources of financial or non-financial support for the overview of reviews, and the role of the funders or sponsors in the overview of reviews. | 22 |
| Competing interests | 25 | Declare any competing interests of the overview of reviews' authors. | 22 |
| Author information | 26a | Provide contact information for the corresponding author. | 1 |
|  | 26b | Describe the contributions of individual authors and identify the guarantor of the overview of reviews. | NA |
| Availability of data and other materials | 27 | Report which of the following are available, where they can be found, and under which conditions they may be accessed: template data collection forms; data collected from included systematic reviews and supplemental primary studies; analytic code; any other materials used in the overview of reviews. | 22 |

**Table S2.** Search strategy.

| **Embase** | #1 'inflammation'/exp OR 'inflammation' OR 'inflammations' OR 'innate inflammatory response' OR 'inflammatory response, innate' OR 'innate inflammatory responses'  #2 'exercise'/exp OR 'exercise' OR 'exercises' OR 'exercise, physical' OR 'exercises, physical' OR 'physical exercise'/exp OR 'physical exercise' OR 'physical exercises' OR 'exercise, aerobic'/exp OR 'exercise, aerobic' OR 'aerobic exercise'/exp OR 'aerobic exercise' OR 'aerobic exercises' OR 'exercises, aerobic' OR 'exercise, isometric'/exp OR 'exercise, isometric' OR 'exercises, isometric' OR 'isometric exercises' OR 'isometric exercise'/exp OR 'isometric exercise' OR 'acute exercise'/exp OR 'acute exercise' OR 'acute exercises' OR 'exercise, acute' OR 'exercises, acute' OR 'exercise training'/exp OR 'exercise training' OR 'exercise trainings' OR 'training, exercise' OR 'trainings, exercise' OR 'physical activity'/exp OR 'physical activity' OR 'activities, physical' OR 'activity, physical'/exp OR 'activity, physical' OR 'physical activities'  #3 meta:ti  #4 #1 AND #2 AND #3  ('inflammation'/exp OR 'inflammation' OR 'inflammations' OR 'innate inflammatory response' OR 'inflammatory response, innate' OR 'innate inflammatory responses') AND ('exercise'/exp OR 'exercise' OR 'exercises' OR 'exercise, physical' OR 'exercises, physical' OR 'physical exercise'/exp OR 'physical exercise' OR 'physical exercises' OR 'exercise, aerobic'/exp OR 'exercise, aerobic' OR 'aerobic exercise'/exp OR 'aerobic exercise' OR 'aerobic exercises' OR 'exercises, aerobic' OR 'exercise, isometric'/exp OR 'exercise, isometric' OR 'exercises, isometric' OR 'isometric exercises' OR 'isometric exercise'/exp OR 'isometric exercise' OR 'acute exercise'/exp OR 'acute exercise' OR 'acute exercises' OR 'exercise, acute' OR 'exercises, acute' OR 'exercise training'/exp OR 'exercise training' OR 'exercise trainings' OR 'training, exercise' OR 'trainings, exercise' OR 'physical activity'/exp OR 'physical activity' OR 'activities, physical' OR 'activity, physical'/exp OR 'activity, physical' OR 'physical activities') AND meta:ti |
| --- | --- |
| **Cochrone library** | #1 Exercise  #2 (Activities, Physical)ti,ab,kw OR (Physical Activities)ti,ab,kw OR (Activity, Physical)ti,ab,kw OR (Physical Activity)ti,ab,kw OR (Exercises, Isometric)ti,ab,kw OR (Exercise, Isometric)ti,ab,kw OR (Isometric Exercises)ti,ab,kw OR (Isometric Exercise)ti,ab,kw OR (Exercise Trainings)ti,ab,kw OR (Exercise Training)ti,ab,kw OR (Trainings, Exercise)ti,ab,kw OR (Training, Exercise)ti,ab,kw OR (Exercise, Aerobic)ti,ab,kw OR (Aerobic Exercise)ti,ab,kw OR (Aerobic Exercises)ti,ab,kw OR (Exercises, Aerobic)ti,ab,kw OR (Acute Exercises)ti,ab,kw OR (Acute Exercise)ti,ab,kw OR (Exercise, Acute)ti,ab,kw OR (Exercises, Acute)ti,ab,kw OR (Physical Exercises)ti,ab,kw OR (Exercise, Physical)ti,ab,kw OR (Exercises, Physical)ti,ab,kw OR (Exercises)ti,ab,kw OR (Physical Exercise)ti,ab,kw  #3 #1 AND #2  #4 Inflammation  #5 (Inflammatory Response, Innate)ti,ab,kw OR (Innate Inflammatory Responses)ti,ab,kw OR (Innate Inflammatory Response)ti,ab,kw OR (Inflammations)ti,ab,kw  #6 #4 AND #5  #7 (meta)ti  #8 #3 AND #6 AND #7 |
| **Pubmed** | ((((((Inflammations[Title/Abstract]) OR (Innate Inflammatory Response[Title/Abstract])) OR (Inflammatory Response, Innate[Title/Abstract])) OR (Innate Inflammatory Responses[Title/Abstract])) OR ("Inflammation"[Mesh])) AND (((((((((((((((((((((((((Exercise, Physical[Title/Abstract]) OR (Exercises, Physical[Title/Abstract])) OR (Physical Exercise[Title/Abstract])) OR (Physical Exercises[Title/Abstract])) OR (Exercise, Aerobic[Title/Abstract])) OR (Aerobic Exercise[Title/Abstract])) OR (Aerobic Exercises[Title/Abstract])) OR (Exercises, Aerobic[Title/Abstract])) OR (Exercise, Isometric[Title/Abstract])) OR (Exercises, Isometric[Title/Abstract])) OR (Isometric Exercises[Title/Abstract])) OR (Isometric Exercise[Title/Abstract])) OR (Acute Exercise[Title/Abstract])) OR (Acute Exercises[Title/Abstract])) OR (Exercise, Acute[Title/Abstract])) OR (Exercises, Acute[Title/Abstract])) OR (Exercise Training[Title/Abstract])) OR (Exercise Trainings[Title/Abstract])) OR (Training, Exercise[Title/Abstract])) OR (Trainings, Exercise[Title/Abstract])) OR (Physical Activity[Title/Abstract])) OR (Activities, Physical[Title/Abstract])) OR (Activity, Physical[Title/Abstract])) OR (Physical Activities[Title/Abstract])) OR ("Exercise"[Mesh]))) AND (meta[Title]) |
| **Web of science** | #1 Topic: 'Inflammations' OR 'Innate Inflammatory Response' OR 'Inflammatory Response, Innate' OR 'Innate Inflammatory Responses' OR 'Inflammation'  #2 Topic: 'exercise' OR 'exercise' OR 'exercises' OR 'exercise, physical' OR 'exercises, physical' OR 'physical exercise' OR 'physical exercise' OR 'physical exercises' OR 'exercise, aerobic' OR 'exercise, aerobic' OR 'aerobic exercise' OR 'aerobic exercise' OR 'aerobic exercises' OR 'exercises, aerobic' OR 'exercise, isometric' OR 'exercise, isometric' OR 'exercises, isometric' OR 'isometric exercises' OR 'isometric exercise' OR 'isometric exercise' OR 'acute exercise' OR 'acute exercise' OR 'acute exercises' OR 'exercise, acute' OR 'exercises, acute' OR 'exercise training' OR 'exercise training' OR 'exercise trainings' OR 'training, exercise' OR 'trainings, exercise' OR 'physical activity' OR 'physical activity' OR 'activities, physical' OR 'activity, physical' OR 'activity, physical' OR 'physical activities'  #3 Title: meta  #4 #1 AND #2 AND #3 |

**Table S3** GRADE classification of quality of evidence

| People with different health conditions | Intervention | Comparation | Outcomes | Included MA | Risk of bias | Inconsistency | Indirectness | Imprecision | Other | Quality |
| --- | --- | --- | --- | --- | --- | --- | --- | --- | --- | --- |
| Postmenopausalwomen | RT | No intervention | CRP | Loaiza-Betancur 2022 | Not serious | Very serious | Not serious | Not serious | None | Moderate |
| Metabolic syndrome | RT | conventional therapy | CRP | Alizaei Yousefabadi 2021 | Very serious | Not serious | Not serious | Very serious | None | Very low |
| Metabolic syndrome | RT | conventional therapy | IL-6 | Alizaei Yousefabadi 2021 | Very serious | Very serious | Not serious | Very serious | None | Very low |
| Metabolic syndrome | RT | conventional therapy | IL-10 | Alizaei Yousefabadi 2021 | Very serious | Not serious | Not serious | Serious | None | Very low |
| Metabolic syndrome | RT | conventional therapy | TNF-α | Alizaei Yousefabadi 2021 | Very serious | Very serious | Not serious | Serious | None | Very low |
| Metabolic syndrome | AE | conventional therapy | CRP | Alizaei Yousefabadi 2021 | Very serious | Very serious | Not serious | Not serious | None | Very low |
| Metabolic syndrome | AE | conventional therapy | IL-6 | Alizaei Yousefabadi 2021 | Very serious | Very serious | Not serious | Serious | None | Very low |
| Metabolic syndrome | AE | conventional therapy | IL-10 | Alizaei Yousefabadi 2021 | Very serious | Serious | Not serious | Serious | None | Very low |
| Metabolic syndrome | AE | conventional therapy | TNF-α | Alizaei Yousefabadi 2021 | Very serious | Very serious | Not serious | Not serious | None | Very low |
| Metabolic syndrome | ART | conventional therapy | CRP | Alizaei Yousefabadi 2021 | Very serious | Not serious | Not serious | Not serious | None | Low |
| Metabolic syndrome | ART | conventional therapy | IL-6 | Alizaei Yousefabadi 2021 | Very serious | Not serious | Not serious | Not serious | None | Low |
| Metabolic syndrome | ART | conventional therapy | IL-10 | Alizaei Yousefabadi 2021 | Very serious | Not serious | Not serious | Not serious | None | Low |
| Metabolic syndrome | ART | conventional therapy | TNF-α | Alizaei Yousefabadi 2021 | Very serious | Not serious | Not serious | Not serious | None | Low |
| Metabolic syndrome | Exercise | conventional therapy | CRP | Alizaei Yousefabadi 2021 | Very serious | Serious | Not serious | Not serious | None | Very low |
| Metabolic syndrome | Exercise | conventional therapy | IL-8 | Alizaei Yousefabadi 2021 | Very serious | Not serious | Not serious | Serious | None | Very low |
| Metabolic syndrome | Exercise | conventional therapy | IL-10 | Alizaei Yousefabadi 2021 | Very serious | Serious | Not serious | Serious | None | Very low |
| Metabolic syndrome | Exercise | conventional therapy | TNF-α | Alizaei Yousefabadi 2021 | Very serious | Very serious | Not serious | Serious | None | Very low |
| Musculoskeletal pain | RT | Low intensity exercise/no exercise | TNF-α | Chi Ngai Lo 2025 | Not serious | Very serious | Not serious | Not serious | None | Moderate |
| Musculoskeletal pain | RT | Low intensity exercise/no exercise | CRP | Chi Ngai Lo 2025 | Not serious | Not serious | Not serious | Not serious | None | High |
| Musculoskeletal pain | RT | Low intensity exercise/no exercise | IL-6 | Chi Ngai Lo 2025 | Not serious | Very serious | Not serious | Serious | None | Low |
| Colorectal cancer | Exercise | conventional therapy | CRP | Hui Zhang 2025 | Not serious | Not serious | Not serious | Serious | None | Moderate |
| Colorectal cancer | Exercise | conventional therapy | IL-6 | Hui Zhang 2025 | Not serious | Serious | Not serious | Very serious | None | Low |
| Colorectal cancer | Exercise | conventional therapy | TNF-α | Hui Zhang 2025 | Not serious | Serious | Not serious | Very serious | None | Low |
| Coronary artery disease | Exercise | conventional therapy | CRP | Gareth Thompson 2020 | Not serious | Very serious | Not serious | Serious | None | Low |
| Coronary artery disease | Exercise | conventional therapy | IL-6 | Gareth Thompson 2020 | Not serious | Serious | Not serious | Serious | None | Moderate |
| Coronary artery disease | Exercise | conventional therapy | IL-8 | Gareth Thompson 2020 | Not serious | Serious | Not serious | Very serious | None | Low |
| Coronary artery disease | Exercise | conventional therapy | IL-10 | Gareth Thompson 2020 | Not serious | Not serious | Not serious | Serious | None | Moderate |
| Coronary artery disease | Exercise | conventional therapy | TNF-α | Gareth Thompson 2020 | Not serious | Not serious | Not serious | Serious | None | Moderate |
| Fibromyalgia | Exercise | No intervention | IL-1B | Luis Suso-Martí 2024 | Not serious | Very serious | Not serious | Very serious | None | Very low |
| Fibromyalgia | Exercise | No intervention | IL-8 | Luis Suso-Martí 2024 | Not serious | Very serious | Not serious | Very serious | None | Very low |
| Fibromyalgia | Exercise | No intervention | TNF-α | Luis Suso-Martí 2024 | Not serious | Very serious | Not serious | Very serious | None | Very low |
| Peripheral Artery Disease | Exercise | No intervention | CRP | Junghoon Lee 2024 | Not serious | Not serious | Not serious | Serious | None | Moderate |
| Peripheral Artery Disease | Exercise | No intervention | IL-6 | Junghoon Lee 2024 | Not serious | Not serious | Not serious | Very serious | None | Low |
| Peripheral Artery Disease | Exercise | No intervention | TNF-α | Junghoon Lee 2024 | Not serious | Not serious | Not serious | Serious | None | Moderate |
| Ankylosing spondylitis | AE | conventional therapy or No intervention | CRP | Conrad Harpham 2022 | Not serious | Not serious | Not serious | Serious | None | Moderate |
| Ankylosing spondylitis | AE | conventional therapy or No intervention | ESR | Conrad Harpham 2022 | Not serious | Not serious | Not serious | Very serious | None | Low |
| Chronic obstructive pulmonary disease | Exercise | conventional therapy | IL-6 | Xiaotian Alex Yue 2024 | Serious | Very serious | Not serious | Serious | None | Very low |
| Chronic obstructive pulmonary disease | Exercise | conventional therapy | CRP | Xiaotian Alex Yue 2024 | Serious | Very serious | Not serious | Serious | None | Very low |
| Chronic obstructive pulmonary disease | Exercise | conventional therapy | TNF-α | Xiaotian Alex Yue 2024 | Serious | Very serious | Not serious | Serious | None | Very low |
| HIV | Exercise | conventional therapy | IL-1β | S. C. Ibeneme 2019 | Serious | Very serious | Not serious | Serious | None | Very low |
| HIV | ART | conventional therapy | IL-6 | S. C. Ibeneme 2019 | Serious | Very serious | Not serious | Very serious | None | Very low |
| HIV | RT | conventional therapy | IL-6 | Hugo R. Zanetti 2021 | Very serious | Very serious | Not serious | Serious | None | Very low |
| HIV | RT | conventional therapy | TNF-α | Hugo R. Zanetti 2021 | Very serious | Serious | Not serious | Very serious | None | Very low |
| Inflammatory bowel disease | Exercise | conventional therapy | IL-6 | Kelly A. Baker 2022 | Not serious | Not serious | Not serious | Very serious | None | Low |
| Inflammatory bowel disease | Exercise | conventional therapy | TNF-α | Kelly A. Baker 2022 | Not serious | Not serious | Not serious | Very serious | None | Low |
| Inflammatory bowel disease | Exercise | conventional therapy | CRP | Kelly A. Baker 2022 | Not serious | Not serious | Not serious | Very serious | None | Low |
| Inflammatory bowel disease | Exercise | conventional therapy | IL-17 | Kelly A. Baker 2022 | Not serious | Not serious | Not serious | Very serious | None | Low |
| Heart Transplant Recipients | AE | conventional therapy | IL-6 | Leandro Tolfo Franzoni 2024 | Not serious | Not serious | Not serious | Serious | None | Moderate |
| Heart Transplant Recipients | AE | conventional therapy | TNF-α | Leandro Tolfo Franzoni 2024 | Not serious | Not serious | Not serious | Very serious | None | Low |
| Heart Transplant Recipients | AE | conventional therapy | adiponectin | Leandro Tolfo Franzoni 2024 | Not serious | Not serious | Not serious | Very serious | None | Low |
| Metabolic disorders | HITT | conventional therapy | adiponectin | Mousa Khalafi 2022 | Not serious | Very serious | Not serious | Serious | None | Low |
| Metabolic disorders | HITT | conventional therapy | Leptin | Mousa Khalafi 2022 | Serious | Serious | Not serious | Very serious | None | Very low |
| Metabolic disorders | HITT | conventional therapy | IL-6 | Mousa Khalafi 2022 | Serious | Not serious | Not serious | Very serious | None | Very low |
| Metabolic disorders | HITT | conventional therapy | TNF-α | Mousa Khalafi 2022 | Serious | Not serious | Not serious | Serious | None | Moderate |
| Metabolic disorders | HITT | conventional therapy | CRP | Mousa Khalafi 2022 | Serious | Not serious | Not serious | Serious | None | Moderate |
| Mild cognitive impairment | Exercise | Low intensity exercise/no exercise | TNF-α | Chuyi Ma 2022 | Serious | Not serious | Not serious | Not serious | None | Moderate |
| Mild cognitive impairment | Exercise | Low intensity exercise/no exercise | IL-1β | Chuyi Ma 2022 | Serious | Not serious | Not serious | Very serious | None | Very low |
| Mild cognitive impairment | Exercise | Low intensity exercise/no exercise | IL-6 | Chuyi Ma 2022 | Serious | Not serious | Not serious | Very serious | None | Very low |
| Mild cognitive impairment | Exercise | Low intensity exercise/no exercise | IL-10 | Chuyi Ma 2022 | Serious | Not serious | Not serious | Very serious | None | Very low |
| Mild cognitive impairment | Exercise | Low intensity exercise/no exercise | CRP | Chuyi Ma 2022 | Serious | Not serious | Not serious | Serious | None | Moderate |
| Mild cognitive impairment | Exercise | Low intensity exercise/no exercise | IFN-γ | Chuyi Ma 2022 | Serious | Not serious | Not serious | Very serious | None | Very low |
| MCI or dementia | Exercise | conventional therapy | TNF-α | Xiuxiu Huang 2021 | Serious | Very serious | Not serious | Very serious | None | Very low |
| MCI or dementia | Exercise | conventional therapy | IL-6 | Xiuxiu Huang 2021 | Serious | Not serious | Not serious | Serious | None | Moderate |
| MCI or dementia | Exercise | conventional therapy | IL-10 | Xiuxiu Huang 2021 | Serious | Serious | Not serious | Very serious | None | Very low |
| MCI or dementia | AE | conventional therapy | TNF-α | Xiuxiu Huang 2021 | Serious | Very serious | Not serious | Very serious | None | Very low |
| MCI or dementia | RT | conventional therapy | TNF-α | Xiuxiu Huang 2021 | Serious | Very serious | Not serious | Very serious | None | Very low |
| MCI or dementia | MBT | conventional therapy | TNF-α | Xiuxiu Huang 2021 | Serious | Very serious | Not serious | Very serious | None | Very low |
| Older adults with sarcopenic obesit | RT | Non-exercise | IL-6 | Huiting Wei 2025 | Serious | Not serious | Not serious | Serious | None | Moderate |
| Older adults with sarcopenic obesit | ART | Non-exercise | IL-6 | Huiting Wei 2025 | Serious | Not serious | Not serious | Serious | None | Moderate |
| Older adults with sarcopenic obesit | Exercise | Non-exercise | TNF-α | Huiting Wei 2025 | Serious | Not serious | Not serious | Serious | None | Moderate |
| Older adults with sarcopenic obesit | Exercise | Non-exercise | CRP | Huiting Wei 2025 | Serious | Not serious | Not serious | Serious | None | Moderate |
| Sarcopenia | RT | conventional therapy | IL-6 | Jingxian Xue 2024 | Serious | Not serious | Not serious | Not serious | None | Moderate |
| Sarcopenia | RT | conventional therapy | TNF-α | Jingxian Xue 2024 | Serious | Not serious | Not serious | Serious | None | Moderate |
| Sarcopenia | RT | conventional therapy | CRP | Jingxian Xue 2024 | Serious | Not serious | Not serious | Serious | None | Moderate |
| Sarcopenia | RT | conventional therapy | IL-10 | Jingxian Xue 2024 | Serious | Serious | Not serious | Serious | None | Very low |
| Adult tobacco smokers | AE | Non-exercise | CRP | Morteza Ghojazadeh 2024 | Not serious | Not serious | Not serious | Serious | None | Moderate |
| Adult tobacco smokers | AE | Non-exercise | TNF-α | Morteza Ghojazadeh 2024 | Not serious | Not serious | Not serious | Serious | None | Moderate |
| Polycystic ovary syndrome (PCOS) | Exercise | Non-exercise | CRP | Mozhgan Hafzi Moori 2023 | Not serious | Very serious | Not serious | Not serious | None | Moderate |
| Polycystic ovary syndrome (PCOS) | Exercise | Non-exercise | adiponectin | Mozhgan Hafzi Moori 2023 | Not serious | Not serious | Not serious | Serious | None | Moderate |
| Multiple sclerosis patients | AE | Non-exercise | IL-6/ TNF-α/IL-10/IFN-γ | V. Bellisario 2024 | Very serious | Serious | Not serious | Not serious | None | Very low |
| Multiple sclerosis patients | Exercise | conventional therapy | IL-6 | Parnian Shobeiri 2022 | Serious | Not serious | Not serious | Serious | None | Moderate |
| Multiple sclerosis patients | Exercise | conventional therapy | TNF-α | Parnian Shobeiri 2022 | Serious | Not serious | Not serious | Serious | None | Moderate |
| Depression | Exercise | Low intensity exercise/no exercise | IL-1B | Maria Eduarda A 2024 | Not serious | Not serious | Not serious | Serious | None | Moderate |
| Depression | Exercise | Low intensity exercise/no exercise | IL-6 | Maria Eduarda A 2024 | Not serious | Serious | Not serious | Serious | None | Moderate |
| Depression | Exercise | Low intensity exercise/no exercise | TNF-α | Maria Eduarda A 2024 | Not serious | Not serious | Not serious | Not serious | None | High |
| Major depression | AE | conventional therapy | IL-6 | Larissa L. da Cunha 2023 | Not serious | Serious | Not serious | Serious | None | Moderate |
| Major depression | AE | conventional therapy | IL-10 | Larissa L. da Cunha 2023 | Not serious | Serious | Not serious | Serious | None | Moderate |
| Major depression | AE | conventional therapy | TNF-α | Larissa L. da Cunha 2023 | Not serious | Not serious | Not serious | Serious | None | Moderate |
| Heart failure | Exercise | conventional therapy | hs-CRP | Abbas Malandish 2023 | Not serious | Serious | Not serious | Not serious | Publication bias | Moderate |
| Heart failure | Exercise | conventional therapy | IL-6 | Abbas Malandish 2023 | Not serious | Not serious | Not serious | Not serious | Publication bias | Very low |
| Heart failure | Exercise | conventional therapy | TNF-α | Abbas Malandish 2023 | Not serious | Very serious | Not serious | Serious | Publication bias | Moderate |
| Heart failure | Exercise | conventional therapy | IL-1β | Abbas Malandish 2023 | Not serious | Not serious | Not serious | Serious | None | Moderate |
| Heart failure | Exercise | conventional therapy | IL-10 | Abbas Malandish 2023 | Not serious | Not serious | Not serious | Serious | None | Moderate |
| Heart failure | Exercise | conventional therapy | IL-18 | Abbas Malandish 2023 | Not serious | Not serious | Not serious | Serious | None | Moderate |
| Rheumatoid arthritis | RT | ROM exercise/conventional therapy | ESR | Zhigang Wen 2021 | Not serious | Very serious | Not serious | Not serious | None | Moderate |
| Rheumatoid arthritis | Exercise | Non-exercise | ESR | Xiaoting Fu 2025 | Not serious | Serious | Not serious | Not serious | None | High |
| Rheumatoid arthritis | Exercise | Non-exercise | CRP | Xiaoting Fu 2025 | Not serious | Not serious | Not serious | Not serious | None | High |
| Rheumatoid arthritis | Exercise | Non-exercise | IL-6 | Xiaoting Fu 2025 | Not serious | Not serious | Not serious | Not serious | None | Moderate |
| Rheumatoid arthritis | Exercise | Non-exercise | TNF-α | Xiaoting Fu 2025 | Not serious | Not serious | Not serious | Serious | None | Moderate |
| Asthma in adults | AE | Conventional therapy | F_ENO_ | Erik Soeren Halvard Hansen 2020 | Not serious | Serious | Not serious | Serious | None | Moderate |
| Asthma in Children | Exercise | Non-exercise | F_ENO_ | Zhenghui Zha 2025 | Not serious | Not serious | Not serious | Serious | None | Very low |
| Cancer survivors | Exercise | Non-exercise | IL-6 | Nasim Khosravi 2019 | Very serious | Not serious | Not serious | Serious | None | Very low |
| Cancer survivors | Exercise | Non-exercise | CRP | Nasim Khosravi 2019 | Very serious | Serious | Not serious | Not serious | None | Very low |
| Cancer survivors | Exercise | Non-exercise | IL-8 | Nasim Khosravi 2019 | Very serious | Serious | Not serious | Serious | None | Very low |
| Cancer survivors | Exercise | Non-exercise | TNF-α | Nasim Khosravi 2019 | Very serious | Serious | Not serious | Serious | None | Low |
| Cancer survivors | Exercise | Non-exercise | IL-1b | Nasim Khosravi 2019 | Very serious | Not serious | Not serious | Not serious | None | Low |
| Cancer survivors | Exercise | Non-exercise | IL-10 | Nasim Khosravi 2019 | Very serious | Not serious | Not serious | Not serious | None | Very low |
| middle-aged and elderly cancer patients | Exercise | Non-exercise | Adiponectin | Jingyu Wang 2025 | Serious | Serious | Not serious | Serious | None | Low |
| middle-aged and elderly cancer patients | Exercise | Non-exercise | Leptin | Jingyu Wang 2025 | Serious | Serious | Not serious | Not serious | None | Low |
| middle-aged and elderly cancer patients | Exercise | Non-exercise | CRP | Jingyu Wang 2025 | Serious | Serious | Not serious | Not serious | None | Very low |
| middle-aged and elderly cancer patients | Exercise | Non-exercise | IL-6 | Jingyu Wang 2025 | Serious | Serious | Not serious | Serious | None | Very low |
| middle-aged and elderly cancer patients | Exercise | Non-exercise | TNF-α | Jingyu Wang 2025 | Serious | Serious | Not serious | Serious | None | Very low |
| middle-aged and elderly cancer patients | Exercise | Non-exercise | IFN-γ | Jingyu Wang 2025 | Serious | Serious | Not serious | Serious | None | Very low |
| middle-aged and elderly cancer patients | Exercise | Non-exercise | IL-8 | Jingyu Wang 2025 | Serious | Serious | Not serious | Serious | None | Very low |
| middle-aged and elderly cancer patients | Exercise | Non-exercise | IL-10 | Jingyu Wang 2025 | Serious | Serious | Not serious | Serious | None | Very low |
| Chronic Kidney Disease | AE | Conventional therapy | CRP | Victor M. Baião 2023 | Serious | Serious | Not serious | Serious | None | Moderate |
| Chronic Kidney Disease | AE | Conventional therapy | IL-6 | Victor M. Baião 2023 | Serious | Not serious | Not serious | Serious | None | Very low |
| Chronic Kidney Disease | AE | Conventional therapy | IL-10 | Victor M. Baião 2023 | Serious | Serious | Not serious | Serious | None | Very low |
| Chronic Kidney Disease | AE | Conventional therapy | TNF-α | Victor M. Baião 2023 | Serious | Serious | Not serious | Serious | None | Moderate |
| Chronic Kidney Disease | RT | Conventional therapy | CRP | Victor M. Baião 2023 | Serious | Not serious | Not serious | Not serious | None | Very low |
| Chronic Kidney Disease | RT | Conventional therapy | IL-6 | Victor M. Baião 2023 | Serious | Serious | Not serious | Serious | None | Very low |
| Chronic Kidney Disease | RT | Conventional therapy | IL-10 | Victor M. Baião 2023 | Serious | Serious | Not serious | Serious | None | Low |
| Chronic Kidney Disease | RT | Conventional therapy | TNF-α | Victor M. Baião 2023 | Serious | Serious | Not serious | Not serious | None | Moderate |
| Chronic Kidney Disease | ART | Conventional therapy | CRP | Victor M. Baião 2023 | Serious | Not serious | Not serious | Serious | None | Moderate |
| Chronic Kidney Disease | ART | Conventional therapy | IL-6 | Victor M. Baião 2023 | Serious | Not serious | Not serious | Serious | None | Moderate |
| Chronic Kidney Disease | ART | Conventional therapy | TNF-α | Victor M. Baião 2023 | Serious | Not serious | Not serious | Serious | None | Moderate |
| Chronic Kidney Disease | Exercise | Conventional therapy | CRP | Victor M. Baião 2023 | Serious | Not serious | Not serious | Not serious | None | Moderate |
| Chronic Kidney Disease | Exercise | Conventional therapy | IL-6 | Victor M. Baião 2023 | Serious | Not serious | Not serious | Not serious | None | Moderate |
| Chronic Kidney Disease | Exercise | Conventional therapy | IL-10 | Victor M. Baião 2023 | Serious | Not serious | Not serious | Serious | None | Moderate |
| Chronic Kidney Disease | Exercise | Conventional therapy | TNF-α | Victor M. Baião 2023 | Serious | Not serious | Not serious | Serious | None | Moderate |
| Chronic Kidney Disease | Exercise | Conventional therapy | CRP | Pawaria 2023 | Serious | Serious | Not serious | Not serious | None | Low |
| Chronic Kidney Disease | Exercise | Conventional therapy | TNF | Pawaria 2023 | Serious | Very serious | Not serious | Serious | None | Very low |
| End-stage kidney disease HD patients | Exercise | Conventional therapy | CRP | Špela Bogataj 2020 | Not serious | Very serious | Not serious | Not serious | None | Very low |
| knee osteoarthritis | AE | Non-exercise | IL-6 | Jenna M Schulz 2020 | Serious | Not serious | Not serious | Serious | None | Very low |
| knee osteoarthritis | Exercise | Non-exercise | CRP | Sofie Puts 2023 | Serious | Not serious | Not serious | Not serious | None | Very low |
| knee osteoarthritis | Exercise | Non-exercise | IL-6 | Sofie Puts 2023 | Serious | Serious | Not serious | Serious | None | Very low |
| knee osteoarthritis | Exercise | Non-exercise | TNF-α | Sofie Puts 2023 | Serious | Serious | Not serious | Very serious | None | Very low |
| Breast Cancer Patients and Survivors with Overweight/Obesity | ART | Non-exercise | IL-6 | Sameer Badri Al-Mhanna 2024 | Very serious | Not serious | Not serious | Serious | None | Very low |
| Breast Cancer Patients and Survivors with Overweight/Obesity | ART | Non-exercise | IL-8 | Sameer Badri Al-Mhanna 2024 | Very serious | Very serious | Not serious | Very serious | None | Very low |
| Breast Cancer Patients and Survivors with Overweight/Obesity | ART | Non-exercise | TNF-α | Sameer Badri Al-Mhanna 2024 | Very serious | Very serious | Not serious | Serious | None | Moderate |
| Breast Cancer Patients and Survivors with Overweight/Obesity | ART | Non-exercise | CRP | Sameer Badri Al-Mhanna 2024 | Very serious | Very serious | Not serious | Very serious | None | Very low |
| Breast Cancer Patients and Survivors  with Overweight/Obesity | Exercise | conventional therapy | CRP | Liang Tan 2025 | Not serious | Very serious | Not serious | Serious | None | Very low |
| Breast Cancer Patients and Survivors with Overweight/Obesity | Exercise | conventional therapy | TNF-α | Liang Tan 2025 | Not serious | Very serious | Not serious | Very serious | None | Very low |
| Breast Cancer Patients and Survivors with Overweight/Obesity | Exercise | conventional therapy | IL-6 | Liang Tan 2025 | Not serious | Very serious | Not serious | Serious | None | Very low |
| Breast Cancer Patients and Survivors with Overweight/Obesity | Exercise | conventional therapy | Leptin | Liang Tan 2025 | Not serious | Very serious | Not serious | Serious | None | Very low |
| Breast Cancer Patients and Survivors with Overweight/Obesity | Exercise | conventional therapy | IL-10 | Liang Tan 2025 | Not serious | Not serious | Not serious | Serious | None | Very low |
| Breast Cancer Patients and Survivors with Overweight/Obesity | Exercise | conventional therapy | Adiponectin | Liang Tan 2025 | Not serious | Very serious | Not serious | Not serious | None | Very low |
| Breast cancer survivors | RT | conventional therapy or Stretching | IL-6 | Francesco Bettariga 2025 | Serious | Not serious | Not serious | Very serious | None | Very low |
| Breast cancer survivors | RT | conventional therapy or Stretching | IL-10 | Francesco Bettariga 2025 | Serious | Not serious | Not serious | Very serious | None | Very low |
| Breast cancer survivors | RT | conventional therapy or Stretching | TNF-α | Francesco Bettariga 2025 | Serious | Not serious | Not serious | Very serious | None | Very low |
| Breast cancer survivors | RT | conventional therapy or Stretching | CRP | Francesco Bettariga 2025 | Serious | Not serious | Not serious | Very serious | None | Very low |
| Breast cancer survivors | AE | conventional therapy or Stretching | IL-6 | Francesco Bettariga 2025 | Serious | Very serious | Not serious | Very serious | None | Very low |
| Breast cancer survivors | AE | conventional therapy or Stretching | IL-8 | Francesco Bettariga 2025 | Serious | Not serious | Not serious | Very serious | None | Very low |
| Breast cancer survivors | AE | conventional therapy or Stretching | IL-10 | Francesco Bettariga 2025 | Serious | Not serious | Not serious | Very serious | None | Very low |
| Breast cancer survivors | AE | conventional therapy or Stretching | TNF-α | Francesco Bettariga 2025 | Serious | Not serious | Not serious | Serious | None | Very low |
| Breast cancer survivors | AE | conventional therapy or Stretching | CRP | Francesco Bettariga 2025 | Serious | Not serious | Not serious | Very serious | None | Very low |
| Breast cancer survivors | ART | conventional therapy or Stretching | IL-6 | Francesco Bettariga 2025 | Serious | Serious | Not serious | Serious | None | Very low |
| Breast cancer survivors | ART | conventional therapy or Stretching | IL-8 | Francesco Bettariga 2025 | Serious | Serious | Not serious | Very serious | None | Very low |
| Breast cancer survivors | ART | conventional therapy or Stretching | IL-10 | Francesco Bettariga 2025 | Serious | Not serious | Not serious | Very serious | None | Very low |
| Breast cancer survivors | ART | conventional therapy or Stretching | TNF-α | Francesco Bettariga 2025 | Serious | Not serious | Not serious | Serious | None | Very low |
| Breast cancer survivors | ART | conventional therapy or Stretching | CRP | Francesco Bettariga 2025 | Serious | Serious | Not serious | Very serious | None | Very low |
| Breast cancer survivors | Exercise | conventional therapy or Stretching | IL-6 | Francesco Bettariga 2025 | Serious | Serious | Not serious | Serious | None | Very low |
| Breast cancer survivors | Exercise | conventional therapy or Stretching | TNF-α | Francesco Bettariga 2025 | Serious | Not serious | Not serious | Serious | Publication bias | Very low |
| Breast cancer survivors | Exercise | conventional therapy or Stretching | CRP | Francesco Bettariga 2025 | Serious | Serious | Not serious | Serious | None | Very low |
| Breast cancer survivors | HIIT+RT | conventional therapy | IL-6 | Yanan Zhou 2022 | Serious | Not serious | Not serious | Not serious | None | Very low |
| Breast cancer survivors | HIIT+AE | conventional therapy | IL-6 | Yanan Zhou 2022 | Serious | Not serious | Not serious | Serious | None | Very low |
| Breast cancer survivors | MBT | conventional therapy | IL-6 | Yanan Zhou 2022 | Serious | Very serious | Not serious | Very serious | None | Very low |
| Type 2 diabetes and overweight/obesity | ART | conventional therapy | CRP | Sameer Badri AL-Mhanna 2024 | Serious | Not serious | Not serious | Serious | None | Very low |
| Type 2 diabetes and overweight/obesity | ART | conventional therapy | TNF-α | Sameer Badri AL-Mhanna 2024 | Serious | Not serious | Not serious | Not serious | None | Very low |
| Type 2 diabetes and overweight/obesity | ART | conventional therapy | IL-6 | Sameer Badri AL-Mhanna 2024 | Serious | Not serious | Not serious | Not serious | None | Very low |
| Type 2 Diabetes Mellitus | RT | conventional therapy | CRP | Rubén Fernández-Rodríguez 2023 | Serious | Not serious | Not serious | Not serious | None | Very low |
| Type 2 Diabetes Mellitus | RT | conventional therapy | TNF-α | Rubén Fernández-Rodríguez 2023 | Serious | Serious | Not serious | Very serious | None | Very low |
| Type 2 Diabetes Mellitus | RT | conventional therapy | IL-6 | Rubén Fernández-Rodríguez 2023 | Serious | Very serious | Not serious | Very serious | None | Very low |
| Type 2 Diabetes Mellitus | AE | Non-exercise | CRP | Georgia Papagianni 2023 | Serious | Very serious | Not serious | Not serious | None | Very low |
| Type 2 Diabetes Mellitus | AE | Non-exercise | IL-6 | Georgia Papagianni 2023 | Serious | Very serious | Not serious | Not serious | None | Very low |
| Type 2 Diabetes Mellitus | AE | Non-exercise | Resistin | Georgia Papagianni 2023 | Serious | Very serious | Not serious | Not serious | Publication bias | Very low |
| Type 2 Diabetes Mellitus | AE | Non-exercise | TNF-α | Georgia Papagianni 2023 | Serious | Very serious | Not serious | Not serious | None | Very low |
| Type 2 Diabetes Mellitus | AE | Non-exercise | Adiponectin | Georgia Papagianni 2023 | Serious | Very serious | Not serious | Serious | None | Very low |
| Type 2 Diabetes Mellitus | ART | Non-exercise | Adiponectin | Keyvan Hejazi 2023 | Serious | Very serious | Not serious | Serious | None | Very low |
| Type 2 Diabetes Mellitus | ART | Non-exercise | Leptin | Keyvan Hejazi 2023 | Serious | Very serious | Not serious | Very serious | None | Very low |
| Type 2 Diabetes Mellitus | ART | Non-exercise | TNF-α | Keyvan Hejazi 2023 | Serious | Very serious | Not serious | Very serious | None | Very low |
| Type 2 Diabetes Mellitus | AE | Non-exercise | CRP | Hayashino 2013 | Serious | Very serious | Not serious | Serious | None | Very low |
| Type 2 Diabetes Mellitus | AE | Non-exercise | TNF | Hayashino 2013 | Serious | Very serious | Not serious | Serious | None | Very low |
| Type 2 Diabetes Mellitus | RT | Non-exercise | CRP | Hejazi 2023 | Serious | Very serious | Not serious | Not serious | None | None |
| Type 2 Diabetes Mellitus | ART | Non-exercise | TNF | Hejazi 2023 | Serious | Very serious | Not serious | Not serious | None | None |
| Type 2 Diabetes Mellitus | ART | Non-exercise | IL-6 | Keyvan Hejazi 2023 | Serious | Very serious | Not serious | Very serious | None | Very low |
| Type 2 Diabetes Mellitus | ART | Non-exercise | CRP | Keyvan Hejazi 2023 | Serious | Very serious | Not serious | Very serious | None | Very low |
| Type 2 Diabetes Mellitus | Exercise | Non-exercise | CRP | Xiaoke Chen | Serious | Very serious | Not serious | Not serious | None | Very low |
| Type 2 Diabetes Mellitus | Exercise | Non-exercise | TNF-α | Xiaoke Chen | Serious | Very serious | Not serious | Not serious | None | Very low |
| Type 2 Diabetes Mellitus | Exercise | Non-exercise | IL-6 | Xiaoke Chen | Serious | Very serious | Not serious | Not serious | None | Very low |
| Middle-Aged and Elderly Patients with T2DM | AE | conventional therapy | CRP | Weijun Yang 2023 | Not serious | Very serious | Not serious | Serious | None | Very low |
| Middle-Aged and Elderly Patients with T2DM | AE | conventional therapy | TNF-α | Weijun Yang 2023 | Not serious | Serious | Not serious | Not serious | None | Low |
| Middle-Aged and Elderly Patients with T2DM | AE | conventional therapy | IL-6 | Weijun Yang 2023 | Not serious | Serious | Not serious | Not serious | None | Moderate |
| Middle-Aged and Elderly Patients with T2DM | AE | conventional therapy | IL-10 | Weijun Yang 2023 | Not serious | Serious | Not serious | Very serious | None | Moderate |
| Middle-Aged and Elderly Patients with T2DM | RT | conventional therapy | CRP | Weijun Yang 2023 | Not serious | Serious | Not serious | Serious | None | Low |
| Middle-Aged and Elderly Patients with T2DM | RT | conventional therapy | TNF-α | Weijun Yang 2023 | Not serious | Very serious | Not serious | Very serious | None | Moderate |
| Middle-Aged and Elderly Patients with T2DM | RT | conventional therapy | IL-6 | Weijun Yang 2023 | Not serious | Serious | Not serious | Very serious | None | Very low |
| Middle-Aged and Elderly Patients with T2DM | RT | conventional therapy | IL-10 | Weijun Yang 2023 | Not serious | Very serious | Not serious | Very serious | None | Low |
| Middle-Aged and Elderly Patients with T2DM | ART | conventional therapy | CRP | Weijun Yang 2023 | Not serious | Very serious | Not serious | Serious | None | Very low |
| Middle-Aged and Elderly Patients with T2DM | ART | conventional therapy | TNF-α | Weijun Yang 2023 | Not serious | Serious | Not serious | Not serious | None | Low |
| Middle-Aged and Elderly Patients with T2DM | ART | conventional therapy | IL-6 | Weijun Yang 2023 | Not serious | Serious | Not serious | Not serious | None | Moderate |
| Middle-Aged and Elderly Patients with T2DM | ART | conventional therapy | IL-10 | Weijun Yang 2023 | Not serious | Not serious | Not serious | Not serious | None | Moderate |
| Middle-Aged and Elderly Patients with T2DM | HIIT | conventional therapy | CRP | Weijun Yang 2023 | Not serious | Not serious | Not serious | Not serious | None | High |
| Middle-Aged and Elderly Patients with T2DM | HIIT | conventional therapy | TNF-α | Weijun Yang 2023 | Not serious | Very serious | Not serious | Very serious | None | High |
| Middle-Aged and Elderly Patients with T2DM | HIIT | conventional therapy | IL-6 | Weijun Yang 2023 | Not serious | Serious | Not serious | Very serious | None | Very low |
| Middle-Aged and Elderly Patients with T2DM | HIIT | conventional therapy | IL-10 | Weijun Yang 2023 | Not serious | Not serious | Not serious | Serious | None | Low |
| Middle-Aged and Elderly Patients with T2DM | Exercise | conventional therapy | CRP | Weijun Yang 2023 | Not serious | Very serious | Not serious | Not serious | None | Moderate |
| Middle-Aged and Elderly Patients with T2DM | Exercise | conventional therapy | TNF-α | Weijun Yang 2023 | Not serious | Serious | Not serious | Not serious | None | Moderate |
| Middle-Aged and Elderly Patients with T2DM | Exercise | conventional therapy | IL-6 | Weijun Yang 2023 | Not serious | Serious | Not serious | Not serious | None | Moderate |
| Middle-Aged and Elderly Patients with T2DM | Exercise | conventional therapy | IL-10 | Weijun Yang 2023 | Not serious | Very serious | Not serious | Serious | None | Moderate |
| Healthy adults | RT | Non-exercise | IL-6 | Miriam Ringleb 2024 | Serious | Very serious | Not serious | Not serious | None | Low |
| Healthy adults | RT | Non-exercise | IL-10 | Miriam Ringleb 2024 | Serious | Very serious | Not serious | Serious | None | Very low |
| Healthy adults | RT | Non-exercise | IL-1ra | Miriam Ringleb 2024 | Serious | Very serious | Not serious | Not serious | None | Very low |
| Healthy adults | RT | Non-exercise | TNF-α | Miriam Ringleb 2024 | Serious | Serious | Not serious | Not serious | None | Very low |
| Healthy adults | RT | Non-exercise | IL-15 | Miriam Ringleb 2024 | Serious | Serious | Not serious | Not serious | None | Low |
| Healthy adults | AE | Non-exercise | IL-6 | Y.-H. WANG 2022 | Not serious | Serious | Not serious | Serious | None | Low |
| Healthy adults | AE | Non-exercise | CRP | Y.-H. WANG 2022 | Not serious | Very serious | Not serious | Very serious | None | Moderate |
| Healthy adults | AE | Non-exercise | TNF-α | Y.-H. WANG 2022 | Not serious | Serious | Not serious | Not serious | None | Very low |
| Healthy adults | Exercise | Non-exercise | hs-CRP | Hammonds 2016 | Serious | Very serious | Not serious | Not serious | None | Low |
| Healthy adults | Exercise | Low intensity exercise/no exercise | IL-6 | Ya-Hai Wang 2023 | Serious | Very serious | Not serious | Not serious | None | Moderate |
| Healthy adults | Exercise | Low intensity exercise/no exercise | CRP | Ya-Hai Wang 2023 | Serious | Very serious | Not serious | Not serious | None | Very low |
| Healthy adults | Exercise | Low intensity exercise/no exercise | TNF-α | Ya-Hai Wang 2023 | Serious | Very serious | Not serious | Not serious | Publication bias | Very low |
| Healthy adults (including Athlete/Soldier) | Exercise | Non-exercise | IL-6 | S.Y. Liu 2025 | Not serious | Not serious | Not serious | Not serious | None | Very low |
| Healthy athletes | AE | Non-exercise | IL-6 | Gashaw Tesema 2019 | Serious | Not serious | Not serious | Not serious | None | High |
| Healthy athletes | AE | Non-exercise | CRP | Gashaw Tesema 2019 | Serious | Not serious | Not serious | Not serious | None | Moderate |
| Healthy Adolescent Athletes | Exercise | Non-exercise | IL-6 | Evdokia Varamenti 2020 | Serious | Very serious | Not serious | Not serious | None | Moderate |
| Healthy Adolescent Athletes | Exercise | Non-exercise | IL-1ra | Evdokia Varamenti 2020 | Serious | Very serious | Not serious | Not serious | None | Very low |
| Healthy Long-Distance Running | AE | Non-exercise | IL-6 | Micael Deivison de Jesus Alves 2022 | Serious | Very serious | Not serious | Not serious | None | Very low |
| Healthy Long-Distance Running | AE | Non-exercise | IL-1ra | Micael Deivison de Jesus Alves 2022 | Serious | Very serious | Not serious | Not serious | None | Very low |
| Healthy Long-Distance Running | AE | Non-exercise | IL-1β | Micael Deivison de Jesus Alves 2022 | Serious | Very serious | Not serious | Not serious | None | Very low |
| Healthy Long-Distance Running | AE | Non-exercise | IL-2 | Micael Deivison de Jesus Alves 2022 | Serious | Very serious | Not serious | Not serious | None | Very low |
| Healthy Long-Distance Running | AE | Non-exercise | IL-4 | Micael Deivison de Jesus Alves 2022 | Serious | Not serious | Not serious | Serious | None | Very low |
| Healthy Long-Distance Running | AE | Non-exercise | IL-8 | Micael Deivison de Jesus Alves 2022 | Serious | Very serious | Not serious | Not serious | None | Moderate |
| Healthy Long-Distance Running | AE | Non-exercise | IL-10 | Micael Deivison de Jesus Alves 2022 | Serious | Very serious | Not serious | Not serious | None | Very low |
| Healthy Long-Distance Running | AE | Non-exercise | TNF-α | Micael Deivison de Jesus Alves 2022 | Serious | Very serious | Not serious | Not serious | None | Very low |
| Healthy Long-Distance Running | AE | Non-exercise | INF-γ | Micael Deivison de Jesus Alves 2022 | Serious | Very serious | Not serious | Not serious | None | Very low |
| Healthy older adults | RT | Non-exercise | CRP | Ali Nejatian Hoseinpour 2025 | Not serious | Very serious | Not serious | Not serious | None | Very low |
| Healthy older adults | RT | Non-exercise | TNF-α | Ali Nejatian Hoseinpour 2025 | Not serious | Very serious | Not serious | Very serious | None | Moderate |
| Healthy older adults | RT | Non-exercise | IL-6 | Ali Nejatian Hoseinpour 2025 | Not serious | Very serious | Not serious | Very serious | None | Very low |
| Healthy Middle-Aged and Older Adults | AE | Non-exercise | IL-6 | Haotian Zhao 2020 | Not serious | Very serious | Not serious | Serious | None | Very low |
| Healthy Middle-Aged and Older Adults | AE | Non-exercise | TNF-α | Haotian Zhao 2020 | Not serious | Very serious | Not serious | Serious | None | Low |
| Healthy Middle-Aged and Older Adults | AE | Non-exercise | CRP | Haotian Zhao 2020 | Not serious | Very serious | Not serious | Serious | None | Low |
| Healthy Middle-Aged and Older Adults | RT | Non-exercise | IL-6 | Haotian Zhao 2020 | Not serious | Serious | Not serious | Serious | None | Low |
| Healthy Middle-Aged and Older Adults | RT | Non-exercise | TNF-α | Haotian Zhao 2020 | Not serious | Very serious | Not serious | Very serious | None | Moderate |
| Healthy Middle-Aged and Older Adults | RT | Non-exercise | CRP | Haotian Zhao 2020 | Not serious | Serious | Not serious | Not serious | None | Very low |
| Healthy Middle-Aged and Older Adults | ART | Non-exercise | IL-6 | Haotian Zhao 2020 | Not serious | Not serious | Not serious | Not serious | None | Low |
| Healthy Middle-Aged and Older Adults | ART | Non-exercise | TNF-α | Haotian Zhao 2020 | Not serious | Not serious | Not serious | Serious | None | High |
| Healthy Middle-Aged and Older Adults | ART | Non-exercise | CRP | Haotian Zhao 2020 | Not serious | Not serious | Not serious | Serious | None | Moderate |
| Healthy Middle-Aged and Older Adults | HIIT | Non-exercise | IL-6 | Haotian Zhao 2020 | Not serious | Not serious | Not serious | Very serious | None | Moderate |
| Healthy Middle-Aged and Older Adults | HIIT | Non-exercise | TNF-α | Haotian Zhao 2020 | Not serious | Not serious | Not serious | Very serious | None | Low |
| Healthy Middle-Aged and Older Adults | HIIT | Non-exercise | CRP | Haotian Zhao 2020 | Not serious | Not serious | Not serious | Serious | None | Low |
| Healthy Middle-Aged and Older Adults | Exercise | Non-exercise | IL-6 | Haotian Zhao 2020 | Not serious | Very serious | Not serious | Not serious | None | Moderate |
| Healthy Middle-Aged and Older Adults | Exercise | Non-exercise | TNF-α | Haotian Zhao 2020 | Not serious | Very serious | Not serious | Not serious | None | Moderate |
| Healthy Middle-Aged and Older Adults | Exercise | Non-exercise | CRP | Haotian Zhao 2020 | Not serious | Very serious | Not serious | Not serious | None | Moderate |
| Older adults | AE | Non-exercise | IL-6 | Seyed Morteza Tayebi 2025 | Serious | Very serious | Not serious | Not serious | None | Moderate |
| Older adults | AE | Non-exercise | CRP | Seyed Morteza Tayebi 2025 | Serious | Very serious | Not serious | Not serious | None | Very low |
| Older adults | AE | Non-exercise | TNF-α | Seyed Morteza Tayebi 2025 | Serious | Very serious | Not serious | Not serious | None | Very low |
| Older adults | AE | Non-exercise | IL-10 | Seyed Morteza Tayebi 2025 | Serious | Very serious | Not serious | Not serious | None | Very low |
| Older adults | Exercise | Non-exercise | IL-6 | Mousa Khalafi | Serious | Not serious | Not serious | Serious | Publication bias | Very low |
| Older adults | Exercise | Non-exercise | TNF-α | Mousa Khalafi | Serious | Not serious | Not serious | Not serious | Publication bias | Very low |
| Older adults | Exercise | Non-exercise | CRP | Mousa Khalafi | Serious | Serious | Not serious | Not serious | None | Low |
| Type 2 diabetes and overweight/obesity | ART | conventional therapy | CRP | Sameer Badri AL-Mhanna 2024 | Serious | Not serious | Not serious | Serious | None | Low |
| Type 2 diabetes and overweight/obesity | ART | conventional therapy | TNF-α | Sameer Badri AL-Mhanna 2024 | Serious | Not serious | Not serious | Not serious | None | Moderate |
| Type 2 diabetes and overweight/obesity | ART | conventional therapy | IL-6 | Sameer Badri AL-Mhanna 2024 | Serious | Not serious | Not serious | Not serious | None | Moderate |
| Breast Cancer Patients and Survivors with Overweight/Obesity | ART | Non-exercise | IL-6 | Sameer Badri Al-Mhanna 2024 | Serious | Not serious | Not serious | Serious | None | Moderate |
| Breast Cancer Patients and Survivors with Overweight/Obesity | ART | Non-exercise | IL-8 | Sameer Badri Al-Mhanna 2024 | Serious | Very serious | Not serious | Very serious | None | Very low |
| Breast Cancer Patients and Survivors with Overweight/Obesity | ART | Non-exercise | TNF-α | Sameer Badri Al-Mhanna 2024 | Serious | Very serious | Not serious | Serious | None | Very low |
| Breast Cancer Patients and Survivors with Overweight/Obesity | ART | Non-exercise | CRP | Sameer Badri Al-Mhanna 2024 | Serious | Very serious | Not serious | Very serious | None | Very low |
| Breast Cancer Patients and Survivors with Overweight/Obesity | Exercise | conventional therapy | CRP | Liang Tan 2025 | Not serious | Very serious | Not serious | Serious | None | Low |
| Breast Cancer Patients and Survivors with Overweight/Obesity | Exercise | conventional therapy | TNF-α | Liang Tan 2025 | Not serious | Very serious | Not serious | Very serious | None | Very low |
| Breast Cancer Patients and Survivors with Overweight/Obesity | Exercise | conventional therapy | IL-6 | Liang Tan 2025 | Not serious | Very serious | Not serious | Serious | None | Low |
| Breast Cancer Patients and Survivors with Overweight/Obesity | Exercise | conventional therapy | Leptin | Liang Tan 2025 | Not serious | Very serious | Not serious | Serious | None | Low |
| Breast Cancer Patients and Survivors with Overweight/Obesity | Exercise | conventional therapy | IL-10 | Liang Tan 2025 | Not serious | Not serious | Not serious | Serious | None | Moderate |
| Breast Cancer Patients and Survivors with Overweight/Obesity | Exercise | conventional therapy | Adiponectin | Liang Tan 2025 | Not serious | Very serious | Not serious | Not serious | None | Moderate |
| Older adults with sarcopenic obesit | RT | Non-exercise | IL-6 | Huiting Wei 2025 | Serious | Not serious | Not serious | Serious | None | Moderate |
| Older adults with sarcopenic obesit | ART | Non-exercise | IL-6 | Huiting Wei 2025 | Serious | Not serious | Not serious | Serious | None | Moderate |
| Older adults with sarcopenic obesit | ART | Non-exercise | TNF-α | Huiting Wei 2025 | Serious | Not serious | Not serious | Serious | None | Moderate |
| Older adults with sarcopenic obesit | ART | Non-exercise | CRP | Huiting Wei 2025 | Serious | Not serious | Not serious | Very serious | None | Very low |
| postmenopausal women  with overweight and obesity: | AE | Non-exercise | CRP | Liang Tan 2023 | Serious | Very serious | Not serious | Not serious | None | Very low |
| postmenopausal women  with overweight and obesity: | AE | Non-exercise | TNF-α | Liang Tan 2023 | Serious | Very serious | Not serious | Not serious | None | Very low |
| postmenopausal women  with overweight and obesity: | AE | Non-exercise | IL-6 | Liang Tan 2023 | Serious | Very serious | Not serious | Not serious | None | Very low |
| postmenopausal women  with overweight and obesity: | AE | Non-exercise | Adiponectin | Liang Tan 2023 | Serious | Very serious | Not serious | Not serious | None | Very low |
| postmenopausal women  with overweight and obesity: | RT | Non-exercise | CRP | Liang Tan 2023 | Serious | Not serious | Not serious | Not serious | None | Moderate |
| postmenopausal women  with overweight and obesity: | RT | Non-exercise | TNF-α | Liang Tan 2023 | Serious | Serious | Not serious | Not serious | None | Low |
| postmenopausal women  with overweight and obesity: | RT | Non-exercise | IL-6 | Liang Tan 2023 | Serious | Not serious | Not serious | Serious | None | Moderate |
| postmenopausal women  with overweight and obesity: | RT | Non-exercise | Adiponectin | Liang Tan 2023 | Serious | Serious | Not serious | Very serious | None | Very low |
| postmenopausal women  with overweight and obesity: | ART | Non-exercise | TNF-α | Liang Tan 2023 | Serious | Serious | Not serious | Not serious | None | Low |
| postmenopausal women  with overweight and obesity: | ART | Non-exercise | IL-6 | Liang Tan 2023 | Serious | Very serious | Not serious | Very serious | None | Very low |
| postmenopausal women  with overweight and obesity: | Exercise | Non-exercise | CRP | Liang Tan 2023 | Serious | Very serious | Not serious | Not serious | None | Very low |
| postmenopausal women  with overweight and obesity: | Exercise | Non-exercise | TNF-α | Liang Tan 2023 | Serious | Very serious | Not serious | Not serious | None | Very low |
| postmenopausal women  with overweight and obesity: | Exercise | Non-exercise | IL-6 | Liang Tan 2023 | Serious | Very serious | Not serious | Not serious | None | Very low |
| postmenopausal women  with overweight and obesity: | Exercise | Non-exercise | Adiponectin | Liang Tan 2023 | Serious | Very serious | Not serious | Not serious | None | Very low |
| overweight/obesity patients with heart failure | Exercise | Non-exercise | TNF-α | Abbas Malandish 2023 | Not serious | Very serious | Not serious | Very serious | None | Very low |
| overweight/obesity patients with heart failure | Exercise | Non-exercise | IL-6 | Abbas Malandish 2023 | Not serious | Not serious | Not serious | Not serious | None | High |
| overweight/obesity patients with heart failure | Exercise | Non-exercise | IL-1β | Abbas Malandish 2023 | Not serious | Serious | Not serious | Very serious | None | Low |
| overweight/obesity patients with heart failure | Exercise | Non-exercise | IL-8 | Abbas Malandish 2023 | Not serious | Serious | Not serious | Very serious | None | Low |
| overweight/obesity patients with heart failure | Exercise | Non-exercise | hs-CRP | Abbas Malandish 2023 | Not serious | Very serious | Not serious | Not serious | Publication bias | Low |
| overweight/obese populations | AE | Non-exercise | IL-1β | Yijian Ding 2022 | Not serious | Serious | Not serious | Not serious | None | Moderate |
| overweight/obese populations | AE | Non-exercise | IL-18 | Yijian Ding 2022 | Not serious | Very serious | Not serious | Very serious | None | Very low |
| overweight/obese populations | RE | Non-exercise | IL-1β | Yijian Ding 2022 | Not serious | Very serious | Not serious | Very serious | None | Very low |
| overweight/obese populations | RT | Non-exercise | IL-18 | Yijian Ding 2022 | Not serious | Not serious | Not serious | Serious | None | Moderate |
| overweight/obese populations | ART | Non-exercise | IL-1β | Yijian Ding 2022 | Not serious | Not serious | Not serious | Not serious | None | High |
| overweight/obese populations | ART | Non-exercise | IL-18 | Yijian Ding 2022 | Not serious | Very serious | Not serious | Very serious | None | Very low |
| overweight/obese populations | HIIT | Non-exercise | IL-18 | Yijian Ding 2022 | Not serious | Not serious | Not serious | Very serious | None | Low |
| overweight/obese populations | Exercise | Non-exercise | IL-1β | Yijian Ding 2022 | Not serious | Serious | Not serious | Not serious | None | Moderate |
| overweight/obese populations | Exercise | Non-exercise | IL-18 | Yijian Ding 2022 | Not serious | Very serious | Not serious | Not serious | None | Moderate |
| Obese children and adolescents | Exercise | Non-exercise | IL-6 | Le-Yang Li 2024 | Serious | Very serious | Not serious | Not serious | None | Very low |
| Obese children and adolescents | Exercise | Non-exercise | TNF-α | Le-Yang Li 2024 | Serious | Very serious | Not serious | Very serious | None | Very low |
| Obese children and adolescents | Exercise | Non-exercise | CRP | Le-Yang Li 2024 | Serious | Serious | Not serious | Not serious | None | Low |
| Obese children and adolescents | Exercise | Non-exercise | Leptin | Le-Yang Li 2024 | Serious | Very serious | Not serious | Not serious | None | Very low |
| Obese children and adolescents | Exercise | Non-exercise | Adiponectin | Le-Yang Li 2024 | Serious | Very serious | Not serious | Not serious | None | Very low |

Abbreviations: AE, Aerobic exercise; RT, Resistance training; ART, Aerobic combined with resistance training; HIIT, High-intensity interval training.
